# Identification and Characterization of Switchgrass Histone *H3* and *CENH3* Genes

**DOI:** 10.3389/fpls.2016.00979

**Published:** 2016-07-12

**Authors:** Jiamin Miao, Taylor Frazier, Linkai Huang, Xinquan Zhang, Bingyu Zhao

**Affiliations:** ^1^Department of Horticulture, Virginia TechBlacksburg, VA, USA; ^2^Department of Grassland Science, Sichuan Agricultural UniversityYa'an, China

**Keywords:** *Panicum virgatum* L., histone H3, CENH3, *Agrobacterium*-mediated transient assay, cell death

## Abstract

Switchgrass is one of the most promising energy crops and only recently has been employed for biofuel production. The draft genome of switchgrass was recently released; however, relatively few switchgrass genes have been functionally characterized. CENH3, the major histone protein found in centromeres, along with canonical H3 and other histones, plays an important role in maintaining genome stability and integrity. Despite their importance, the histone *H3* genes of switchgrass have remained largely uninvestigated. In this study, we identified 17 putative switchgrass histone *H3* genes *in silico*. Of these genes, 15 showed strong homology to histone *H3* genes including six *H3.1* genes, three *H3.3* genes, four *H3.3-like* genes and two *H3.1-like* genes. The remaining two genes were found to be homologous to *CENH3*. RNA-seq data derived from lowland cultivar Alamo and upland cultivar Dacotah allowed us to identify SNPs in the histone *H3* genes and compare their differential gene expression. Interestingly, we also found that overexpression of switchgrass histone *H3* and *CENH3* genes in *N. benthamiana* could trigger cell death of the transformed plant cells. Localization and deletion analyses of the histone *H3* and *CENH3* genes revealed that nuclear localization of the N-terminal tail is essential and sufficient for triggering the cell death phenotype. Our results deliver insight into the mechanisms underlying the histone-triggered cell death phenotype and provide a foundation for further studying the variations of the histone *H3* and *CENH3* genes in switchgrass.

## Introduction

Plant nucleosomes are composed of a protein octamer that contains two molecules of each of the core histone proteins: H2A, H2B, H3, and H4. The canonical histone H3 protein is one of the most important protein components of this complex. The histone H3 protein has a main globular domain (including αN-helix, α1-helix, Loop1, α2-helix, Loop2, and α3-helix motifs), a long N-terminal tail and is post-translationally modified significantly more than the other histone proteins (Ingouff and Berger, 2010). The histone H3 family consists of four main members. Three of these members, including H3.1, H3.2, and H3.3, share more than 95% identity. The fourth member consists of the histone H3 variant CENH3, which is highly divergent in sequence structure in comparison to canonical histone H3. CENH3 is specifically present at centromeres and is essential for centromere and kinetochore formation in all organisms (Allshire and Karpen, 2008).

The histone H3 proteins have been extensively characterized in many plant species. Recent studies have demonstrated that histone H3 variants have evolved to play an important role in specialized plant functions, including heterochromatin replication (Jacob et al., 2014), gene silencing (Mozzetta et al., 2014), flowering time regulation (Shafiq et al., 2014), stem elongation (Chen et al., 2013), gene activation (Ahmad and Henikoff, 2002a,b) and so on. More recently, some studies have reported that the aberrant expression of *CENH3* may cause errors during mitosis, such as triggering the formation of micronuclei and the subsequent loss of chromosomes, thus resulting in aneuploidy and possibly reduced fertility (Ravi and Chan, 2010; Lermontova et al., 2011; Sanei et al., 2011).

Switchgrass (*Panicum virgatum* L.), a warm-season perennial grass species native to North America, has a growth habitat that ranges from northern Mexico into southern Canada. Switchgrass has recently been targeted as a model herbaceous species for biofuel feedstock development (McLaughlin and Adams Kszos, 2005). With a base chromosome number of 9, ploidy levels within naturally occurring populations of the species vary from diploid (2n = 2x = 18) to dodecaploid (2n = 12x = 108) (Nielsen, 1944; Hultquist et al., 1996). Switchgrass has two distinct ecotypes, lowland and upland, that are characterized based on growth habitat. The lowland ecotypes, which are commonly tetraploid (2n = 4x = 36), are frequently found in warm and humid environments in the southern regions of North America. The upland ecotypes, however, can vary in ploidy (4*X*, 6*X*, and 8*X*) and are adapted to colder and more arid environments in central to northern North America (Porter, 1966; Hopkins et al., 1996). Under natural conditions, inter-ecotype hybridizations between switchgrass species with different ploidy levels are rare. Even so, aneuploidy is common in switchgrass, indicating that its genome is unstable in nature and making it difficult for switchgrass genetic studies and breeding selection (Costich et al., 2010). The cause of switchgrass genome instability could be related to abnormal centromeres that interact with the mitotic spindle, which can lead to selective chromosome loss (Bennett et al., 1976). It is possible that preferential or differential expression of switchgrass *H3* and *CENH3* genes may contribute to switchgrass genome instability. Despite their importance, both switchgrass histone *H3* and *CENH3* genes have remained largely uninvestigated.

In this study, we used a homology based approach to identify histone *H3* and *CENH3* genes in switchgrass. We identified 15 putative switchgrass histone *H3* genes and two *CENH3* genes. RNA-seq analysis allowed us to detect genetic polymorphisms in these genes, as well as differences in their expression levels, between upland cultivar Alamo and lowland cultivar Dacotah. We also characterized these switchgrass histone *H3* and *CENH3* genes with respect to their subcellular localization and phenotypic responses in *Nicotiana benthamiana*. Interestingly, we report for the first time that transient overexpression of histone *H3* or *CENH3* genes can trigger programmed cell death in *Nicotiana benthamiana*. The results of this study provide a better understanding of the function of switchgrass histone *H3* genes and may aid in improving future genetic studies of this important biofuel crop.

## Materials and methods

### Plant material

Switchgrass cv. Alamo, cv. Dacotah and *N. benthamiana* (PI 555478) seeds were obtained from the USDA (United States Department of Agriculture) germplasm center. Italian ryegrass (*Lolium multifolorum* L.) cv. Changjiang No.2 seeds were obtained from Sichuan Agricultural University of China. Two independent *NtSGT1*-RNAi *N. tabacum* transgenic lines (Traore et al., under revision) were also used for histone *H3* transient expression analyses. Switchgrass, tobacco and Italian ryegrass were planted in pots and grown in a growth chamber programmed for 16 h light at 28°C and 8 h dark at 24°C.

### Database search and sequence analysis

The Arabidopsis histone *H3* gene (At1g09200) was used as a query to BLASTX search against the switchgrass draft genome (*Panicum virgatum* v1.1, DOE-JGI, http://www.phytozome.net/pvirgatum). Multiple sequence alignment of histone H3 genes/proteins was performed using DNAMAN 7.0 (Lynnon Biosoft, San Ramon, USA), and visualized using BOXSHADE 3.21 (http://www.ch.embnet.org/software/BOX_form.html). The neighbor-joining phylogenetic tree was generated using MEGA6 (Tamura et al., 2013).

### RNA-seq analysis of the putative switchgrass histone *H3* and *CENH3* genes

RNA-sequencing reads from leaf tissue of three biological replicates of Alamo and Dacotah were obtained (Hupalo et al., in preparation) and imported into CLC Genomics Workbench version 7.5 (CLCBio/Qiagen, Boston, MA). The reads were quality trimmed and filtered and mapped to the *Panicum virgatum* reference genome (v1.1) using the default parameters with the following adjustments: maximum number of hits for a read = 1, similarity fraction = 0.9, length fraction = 1.0, mismatch cost = 2, insertion cost = 3 and deletion cost = 3. After mapping, the reads were locally realigned and variants were called using the Basic Variant Detection tool with the following settings: ploidy = 4, minimum coverage = 3, minimum count = 2, minimum frequency (%) = 25.0 and base quality filter = yes. The RPKM and fold difference values for the switchgrass histone *H3* and *CENH3* genes of Alamo and Dacotah were calculated using an unpaired two-group comparison experiment feature that is part of the CLC Genomics Workbench program.

### RT-PCR analysis of the histone *H3* genes

Total RNAs from fresh leaves of Alamo, tobacco, Arabidopsis and Italian ryegrass were isolated using a RNeasy® Plant Mini Kit (Qiagen, Valencia, CA) and were treated with the RNase-Free DNase Set (Qiagen) to remove DNA contamination. Reverse transcription (RT) of the first strand cDNA synthesis was performed using a DyNAmo™ cDNA Synthesis Kit (Fisher Scientific Inc, Pittsburgh, PA). PCR was performed using either gene-specific primers or conserved primers, as listed in Table S1. Gene-specific primers were designed based on the nucleotide sequences of the 5′ and 3′ UTRs of specific switchgrass histone *H3* genes. Switchgrass histone *H3* conserved primers were designed based on the sequence alignment of the 15 switchgrass *histone H3* genes. Tobacco and Italian ryegrass histone *H3* gene-specific primers were designed based on known ESTs (EF051133.1, AB366152.1, and AB205017.1) located in GenBank. Primers for *Arabidopsis thaliana H3.3* (At1g13370) and *CENH3* (At1g01370) were designed based on the nucleotide sequences identified in the TAIR (The Arabidopsis Information Resource) database.

The iProof™ high fidelity DNA polymerase (Bio-Rad, Hercules, CA) was used for all PCR amplifications and the PCR program was run as follows: 98°C for 3 min; 98°C for 30 s, 58°C for 45 s, 72°C for 50 s (30 cycles); and a final extension at 72°C for 7 min. The PCR products were gel purified using an E.Z.N.A.® MicroElute Gel Extraction Kit (Omega Bio-Tek Inc., Norcross, GA) and were subsequently cloned into the pENTR^TM^/D-TOPO® vector (Invitrogen, Carlsbad, CA). The vector was then transformed into *E. coli* (*DH5*α) (Life Technologies) and the bacteria were grown at 37°C. Plasmid DNAs were isolated using a Qiagen plasmid miniprep kit (Qiagen, Valencia, CA) and sequenced using a M13 forward primer (Table S1) at the core facility of Virginia Bioinformatics Institute (Blacksburg, VA, USA).

For deletion analyses of switchgrass histone *H3* genes, a series of primers (Table S1, **Figure 3**) were designed to amplify different fragments based on the gene sequence of *PvH3.3* (Pavir.Ib01857.1) and *PvCENH3* (Pavir.J05674.2). Chimeric gene fusions of the *PvH3.3* N-terminal tail and the *PvCENH3* fold-domain were constructed by overlapping PCR (**Figure 3C**).

### Cloning of the histone *H3* genes into a plant expression vector

Using the Gateway®LR Clonase® II Enzyme mix kit (Invitrogen, Carlsbad, CA), the different histone *H3* genes were sub-cloned into the binary vectors pEarleyGate101, pEarleyGate104 (Earley et al., 2006) and pEAQ-HT-DEST3 (Sainsbury et al., 2009). These vectors were then transformed into *E. coli* (*C2110*) (Wu and Zhao, 2013) by electroporation. The derived histone *H3-YFP, YFP-H3*, and *H3*-Hisx6 fusion genes were cloned behind the CaMV35S promoter. The plasmid constructs were sequenced using either the 35S forward primer, the YFP forward primer or gene-specific primers (Table S1).

### *Agrobacterium*-mediated transient assays in tobacco plants

The pEarleyGate101-PvH3s-YFP, pEarleyGate104-YFP-H3s, and pEAQ-HT-PvH3.3-Hisx6 plasmid DNAs were transformed into *Agrobacterium tumefaciens* strain *GV2260* by electroporation. Transformed *Agrobacterium* cells were grown on LB agar medium supplemented with kanamycin 50 μg/ml and rifampicin 100 μg/ml and incubated at 28°C for 2 days. *Agrobacterium*-mediated transient assays in tobacco leaves were performed as described previously (Traore and Zhao, 2011). The fully expanded leaves of 3–4 week old *N. benthamiana* or *N. tabacum* plants were chosen for infiltration. The fluorescence signal of the histone H3-YFP protein was monitored 2 days post inoculation using a confocal microscope (Zeiss Axio Observer.A1, Carl Zeiss MicroImaging, Inc., Thornwood, NY).

### Analysis of histone H3-YFP fusion proteins by western blot

*Agrobacterium tumefaciens GV2260* cultures carrying the pEG101-PvH3s-YFP constructs were infiltrated into young but fully expanded tobacco leaves at a concentration of OD_600nm_ = 0.5. Leaf disks (1.9 cm diameter) were collected 3 days post inoculation using a cork borer, ground in liquid nitrogen and re-suspended in 100 μl 3 × Laemmli buffer containing 16% β-mercaptoethanol. The tissue was then boiled for 10 min and pelleted at a high speed for 10 min. Twenty micro liters of protein extract was applied to and separated on a 10% SDS-PAGE gel. The proteins were blotted to a PVDF membrane using a Bio-Rad Trans-Blot®Turbo^TM^ Transfer System. The membrane was blocked with 5% nonfat skim milk in 1 × Tris-saline buffer supplemented with 0.5% Tween 20 (1 × TBST). Next, the membrane was probed with anti-HA-HRP (Abcam, 1:2000) and the signal was detected with using SuperSignal® West Pico Chemiluminescent Substrate (Thermo Scientific, Waltham, MA). The chemiluminescent signals were exposed to autoradiography film (Genesee Scientific, San Diego, CA) using a Kodak film processor (Kodak, A Walsh Imaging, Inc, Pompton Lakes, NJ).

## Results

### Seventeen histone *H3* genes were identified from the current draft switchgrass genome

To identify putative switchgrass histone *H3* genes, we used the Arabidopsis histone *H3* gene (At1g09200) as a query to BLASTx search against the switchgrass draft genome (*Panicum virgatum* v1.1). Seventeen potential switchgrass histone *H3* genes, which showed significant similarity to the Arabidopsis histone *H3* gene, (the majority of *E*-values were < 1.00e-30) were identified (Table 1).

**Table 1:** **Summary of database search and expression analysis for 17 Switchgrass ***Histone H3*** genes**.

**Protein ID**	**Gene ID (nt)^a^**	**H3 type**	**E-value^b^**	**Alamo RPKM ^c^ (mean)**	**Dacotah RPKM ^c^ (mean)**	**Fold Difference^d^**	**Length (aa)**	**Number of introns**	**Chromosome Location^e^**	**Number of SNPs between Alamo and Dacotah**
PvCENH3.1	Pavir.J05674.2	CENH3	1.00E-03	0.43	0.57	1.17	167	6	contig08989	8
PvCENH3.2	Pavir.J25829.1	CENH3	0.18	0.33	0.98	2.97	167	6	contig28575	0
PvH3.1	Pavir.Db02133.1	H3.1	9.00E-41	0	0	−	136	0	Chr04b	0
	Pavir.J20671.1	H3.1	8.00E-32	0.06	0.14	2.48	136	0	contig224856	0
	Pavir.Ga01868.1	H3.1	2.00E-38	0	0.04	−	136	0	Chr07a	0
	Pavir.J18804.1	H3.1	3.00E-31	0	0	−	136	0	contig20530	0
	Pavir.J00640.1	H3.1	6.00E-48	1.95	0.07	−29.22	136	0	contig00653	4
	Pavir.J01005.1	H3.1	1.00E-49	5.61	0.51	−10.89	136	0	contig01099	14
PvH3.1-like-A	Pavir.Fa02085.1	H3.1-like	2.00E-41	0	0.07	−	137	0	Chr06a	0
PvH3.1-like-B	Pavir.J07529.1	H3.1-like	6.00E-45	0	0	−	136	0	contig110228	0
PvH3.3	Pavir.Ia03121.2	H3.3	3.00E-93	406.03	494.74	1.22	136	3	Chr09a	6
	Pavir.Ib01857.1	H3.3	1.00E-95	670.22	514.42	−1.3	136	3	Chr09b	15
	Pavir.J05563.1	H3.3	8.00E-35	11.89	26.73	2.25	136	1	contig08697	24
PvH3.3-like-A	Pavir.J26857.1	H3.3-like	1.00E-95	49.51	32.06	−1.54	136	3	contig30080	11
	Pavir.J24812.1	H3.3-like	5.00E-98	100.67	88.05	−1.14	136	3	contig27272	25
PvH3.3-like-B	Pavir.J09299.1	H3.3-like	3.00E-93	1.17	1.96	1.67	136	3	contig123174	16
PvH3.3-like-C	Pavir.J10481.1	H3.3-like	1.00E-55	0.04	0	−	136	2	contig131996	0

a *Full length genes were found at these locations in the Phytozome v 10.1 Panicum virgatum v1.1 genome*.

b *E-value are shown in this table as determined by a TAIR CDS BLAST search using switchgrass histone H3 nucleotide sequences*.

c *RPKM = reads per kilobase per million = Number of mapped reads/length of transcript in kilo base/million mapped reads*.

d *Fold Difference = the amount of the mean expression values differ between Alamo and Dacotah*.

e *Chromosome Location can find in Phytozome v10.1 Panicum virgatum v1.1 genome*.

**Figure 1:**
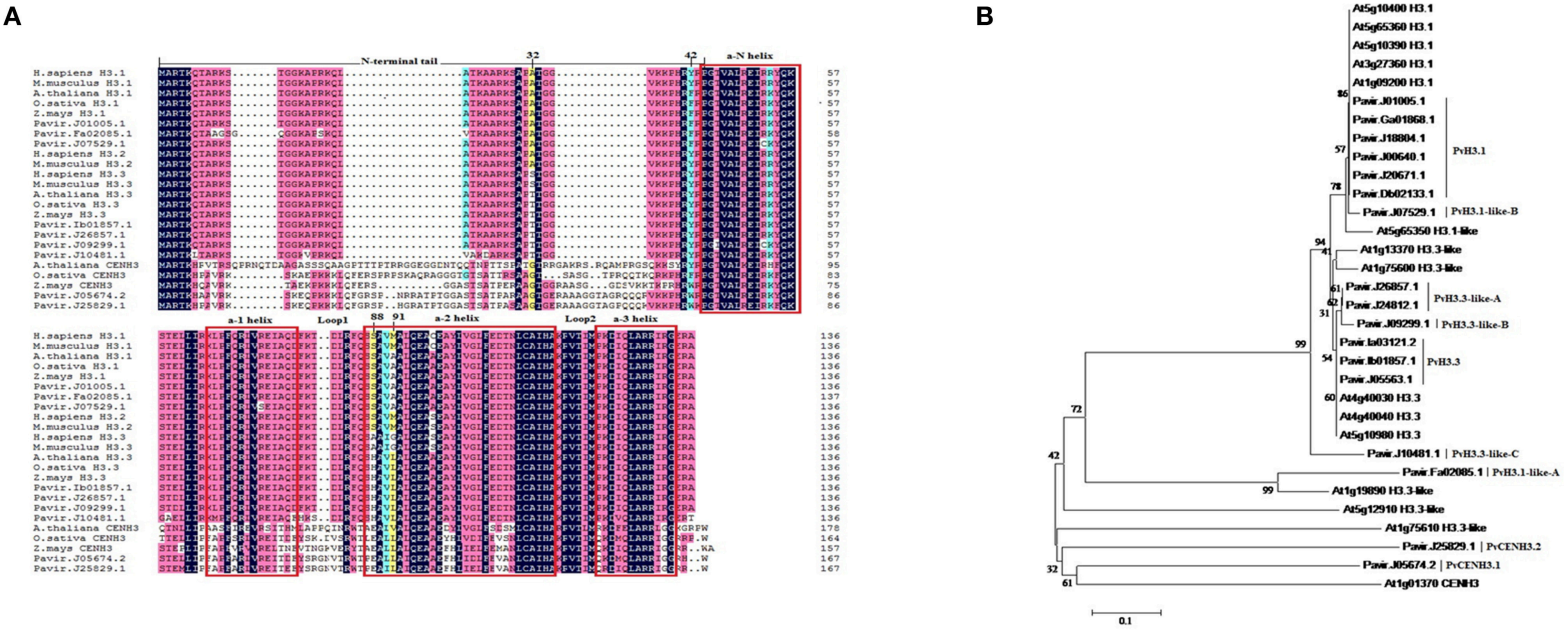
**Alignment and phylogenetic analysis of the protein sequences of switchgrass histone H3 and histone H3 from other organisms. (A)** Multiple alignments between switchgrass histone H3s and other species. Four amino acid positions (32, 42, 88, 91) that are different between H3.1 and H3.3 are labeled. Red boxes indicate helices within histone fold domain; **(B)** A neighbor-joining tree of switchgrass and Arabidopsis histone H3s.

The seventeen switchgrass histone *H3* genes encode nine different proteins (Table 1). These nine proteins were aligned with human (NCBI Reference Sequence: NP_003520.1, NP_066403.2, and NP_002098.1), mouse (NP_659539.1, NP_038576.1, and NP_032236.1), Arabidopsis (At1g09200, At4g40030, and At1g01370), rice (Os01g0866200, Os03g0390600, and Os05g0489800) and maize (NP_001131276.1, AFW71933.1, and NP_001105520.1) histone H3 and CENH3 proteins (Figure 1A). All of the switchgrass histone H3 proteins, except for Pavir.J05674.2 and Pavir.J25829.1, which encode putative CENH3 proteins, display a high degree of homology and conservation to the histone proteins of these different species.

To further classify the switchgrass histone H3 proteins, we used the switchgrass and Arabidopsis histone H3 proteins to generate a phylogenetic tree (Figure 1B). The switchgrass histone H3 proteins largely grouped into five clades. The first clade consisted of Pavir.J01005.1, Pavir.Ga01868.1, Pavir.J18804.1, Pavir.J00640.1, Pavir.J20671.1, and Pavir.Db02133.1 and grouped with the Arabidopsis H3.1 proteins. The second clade contained Pavir.J07529.1, which grouped with the Arabidopsis H3.1-like proteins. The third clade consisted of Pavir.J26857.1, Pavir.J24812.1 and Pavir.J09299.1 and grouped with the Arabidopsis H3.3-like proteins. The fourth clade contained Pavir.Ia03121.2, Pavir.Ib01857.1, and Pavir.J05563.1 and grouped with the Arabidopsis H3.3 proteins. Finally, Pavir.J05674.2 and Pavir.J25829.1 grouped with the Arabidopsis CENH3 protein. Pavir.Fa02085.1 and Pavir.J10481.1, along with the H3.1-like and H3.3-like proteins, are novel H3 variants; however, they appear to be clustered into the H3.3 or H3.1 groups due to amino acid substitutions commonly found in these variants at positions 32, 42, 88, and 91 (Luger et al., 1997; Malik and Henikoff, 2003). These proteins also contain other sequence variations, such as insertions. Corresponding to which Arabidopsis proteins they grouped closely to, we renamed these switchgrass histone H3 proteins as PvH3.1, PvH3.1-like (A and B), PvH3.3, PvH3.3-like (A, B, and C), PvCENH3.1, and PvCENH3.2. Therefore, we identified six major histone H3s (H3.1) and three histone H3 variants (H3.3) from the current draft switchgrass genome (Figure 1, Table 1).

Although, most of the canonical switchgrass histone H3 and H3 variants have 136 amino acids, we also found histone H3 variants (Pavir.Fa02085.1) that have small insertions at position 12. Pavir.J05674.2 and Pavir.J25829.1 are centromeric histone H3 variants (PvCENH3) that have highly diverse sequences. CENH3 shows significant sequence divergence among both switchgrass and Arabidopsis, and it even differs significantly from canonical histone H3s, which are highly variable in the N-terminal tail; however, the histone fold domain is relatively conserved (Figure 1).

Using the gene IDs, the CDS sequences for all of the switchgrass histone *H3* genes were obtained from Phytozome (*Panicum virgatum* v1.1). Alignment of the CDS sequences with the genomic DNA sequences for all of the switchgrass histone *H3* genes allowed us to identify the exons and introns of these genes. The *PvH3.1* genes do not have any introns whereas the *PvH3.3* and *PvCENH3* contain between 1–6 introns (Table 1). Although the intron splicing sites are conserved, the sequence content in the introns is highly diverse (Figure S1).

### Sequence polymorphism and expression variation of histone *H3* genes identified from two switchgrass cultivars

In order to evaluate gene expression of the 17 putative switchgrass histone *H3* genes, we obtained and analyzed RNA-seq datasets that were constructed from the leaf tissue of two switchgrass cultivars, Alamo and Dacotah (Hupalo et al., in preparation). As summarized in Table 1, at least 10 of the histone *H3* genes could be identified in the RNA-seq datasets that were generated from Alamo and Dacotah. Based on the RPKM (Reads Per Kilobase per Million reads mapped) values, four of the switchgrass histone *H3* genes (Pavir.J26857.1, Pavir.J24812.1, Pavir.Ia03121.2, and Pavir.Ib01857.1) are expressed at relatively higher levels than the others in switchgrass leaf tissue.

The expression variation of the Alamo and Dacotah histone *H3* genes was also analyzed by comparing the expression fold difference values between the two cultivars. As shown in Table 1, four out of the 17 histone *H3* genes displayed more than a 2-fold expression difference in RPKM values. Interestingly, two Alamo histone *H3.1* genes (Pavir.J00640.1 and Pavir.J01005.1) are expressed at 29.22 and 10.89 folds higher, respectively, than their homologous genes in Dacotah.

The current release of the switchgrass genome (*Panicum virgatum* v1.1) contains 636 Mb of sequencing data assembled onto 18 scaffolds with an additional 593 Mb remaining on unanchored contigs (http://phytozome.jgi.doe.gov). Five of the 17 histone *H3* genes identified in this study (Pavir.Fa02085.1, Pavir.Db02133.1, Pavir.Ga01 868.1, Pavir.Ia03121.2, and Pavir.Ib01857.1) have been anchored onto a given switchgrass chromosome, while the others are located on contigs. Eight out of the 17 histone *H3* genes are identical in Alamo and Dacotah. The other nine genes have nucleotide sequence polymorphisms that contain anywhere from 4 to 25 SNPs (Table 1, Table S2).

### Cloning switchgrass histone *H3* genes by RT-PCR

To validate the gene sequences and expression levels of the predicted switchgrass histone H3 genes, we used a pair of conserved primers (Table S1) to perform RT-PCR on the leaf cDNAs of cv. Alamo. PCR products were cloned and 22 clones were randomly chosen for sequencing analysis. Fifteen clones (68%) carried the DNA sequences coding for *PvH3.3*, which suggests that the *PvH3.3* gene has a relatively higher expression level in switchgrass leaf tissue in comparison to the other histone *H3* genes. This is consistent with the RNA-seq data in which the two *PvH3.3* genes in both Alamo and Dacotah exhibited a relatively higher RPKM value than the RPKM value of the other histone genes (Table 1).

Four *H3.1* genes (Pavir.Ga01868.1, Pavir.J18804.1, Pavir.J00640.1, and Pavir.J01005.1), two *H3.3* genes (Pavir.Ia03121.2 and Pavir.Ib01857.1), two *H3.3-like* genes (Pavir.J26857.1 and Pavir.J24812.1) and one *CENH3* gene (Pavir.J05674.2) were also amplified from Alamo cDNA using the histone *H3* and *CENH3* specific primers (Table S1). In addition, one *H3.3* gene (*PvH3.3*) was amplified in our RT-PCR analysis (data not shown) that was not identified in the switchgrass genome (*Panicum virgatum* v1.1).

### PvH3s and pvCENH3 fused to YFP are predominately located in the plant cell nucleus

To test the subcellular localization of the switchgrass histone *H3* genes, we cloned Pavi r.J01005.1 (*PvH3.1*), Pavir.Ib01857.1 (*PvH3.3*), Pavir.J24812.1 (*PvH3.3-like*), Pavir.J05674.2 (*PvCENH3*), and *PvH3.3* (not identified in our genome search, but identified by RT-PCR) into the binary vector pEarleygate101 (Earley et al., 2006), which fused a C-terminus YFP (yellow fluorescent protein) to each of the histone genes. The fusion genes were then transiently expressed in *Nicotiana benthamiana* plant cells. PvH3s-YFP and PvCENH3-YFP fusion proteins localized predominantly in the plant cell nucleus, whereas the control YFP was located in both the cytosol and the nucleus (Figure S2).

### Overexpression of histone *H3-YFP* triggers a cell death phenotype in *N. benthamiana*

**Figure 2:**
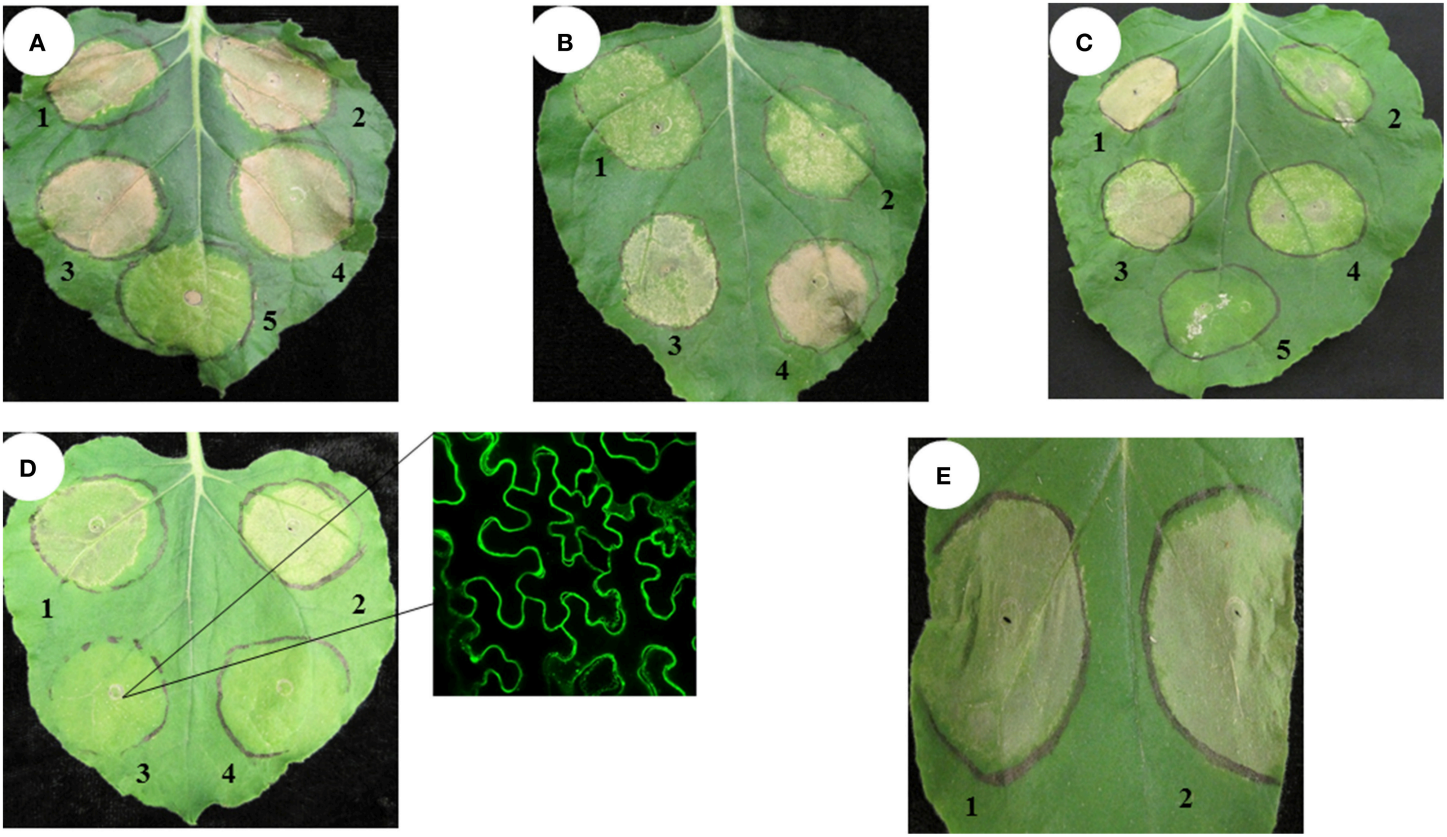
**Transient expression of switchgrass ***histone H3*** in ***N. benthamiana*** triggered cell death phenotype**. Agrobacterium strains (OD_600_ = 0.5) expressing different constructs were inoculated in tobacco leaves. The cell death phenotype was pictured at 4–7 days post inoculation. Pv, Switchgrass; At, Arabidopsis; Nb, tobacco; Lm, ryegrass. **(A)** 1. Pavir.J01005.1-YFP; 2. Pavir.Ib01857.1-YFP; 3. Pavir.J24812.1-YFP; 4. PvH3.3-YFP; 5. YFP only (negative control); **(B)** 1. Pavir.J05674.2-YFP; 2. AtCENH3-YFP; 3. AtH3.3-YFP; 4. LmH3.3-YFP; **(C)** 1. NbH3.3-YFP; 2. NbCENH3-YFP; 3. YFP-Pavir.Ib01857.1; 4. YFP-Pavir.J05674.2; 5. YFP only; **(D)** 1. Pavir.J05674.2-YFP; 2.Pavir.Ib01857.1-YFP; 3. MyrYFP-Pavir.Ib0185 7.1; 4. YFP only; the right one is MyrYFP-Pavir.Ib01857.1 (YFP fluorescence signal was predominately localized in the plasma membrane of transformed plant cells); **(E)** 1. Pavir.Ib01857.1–6xHistidine; 2. Pavir.Ib01857.1-YFP.

When transiently overexpressed in *N. benthamiana*, both the PvH3-YFP and the PvCENH3-YFP proteins triggered a cell death phenotype 3 days post inoculation (dpi) (Figures 2A,B). Interestingly, overexpression of the histone *H3* genes cloned from other plant species, including Arabidopsis *H3.3* and *CENH3*, Italian ryegrass *H3.3* and *N. benthamiana H3.3* and *CENH3*, also triggered cell death in the transformed *N. benthamiana* plant cells (Figures 2B,C).

To test if the C-terminal YFP fusion affected the cell-death-triggering ability of histone *H3*, we fused YFP to the N-terminus of switchgrass H3.3 by cloning the *H3.3* gene into the binary vector pEarleygate 104 (Earley et al., 2006). As shown in Figure 2C, YFP-PvH3.3 and YFP-PvCENH3 could also trigger cell death.

Fusion of YFP to either end of a protein may alter the native protein structure and function. Thus, YFP-tagged proteins can produce unexpected and unwanted phenotypes. To determine if YFP was contributing to the cell death phenotype of the histone *H3* genes, we also cloned *PvH3.3* into the pEAQ-HT-DEST3 vector, which fuses a smaller tag (6xHistidine) to the C-terminal end of the protein (Sainsbury et al., 2009). As shown in Figure 2E, transient expression of pEAQ-HT-PvH3.3 was still able to trigger cell death. Therefore, we conclude that overexpression of solely the PvH3.3 protein is able to trigger cell death in *N. benthamiana*.

Histone H3 is one of the key elements of the nucleosome, which is predominately located in the nucleus. To test if nuclear localization of the histone H3s is required for its ability to trigger the cell death phenotype, we fused a myristoylation signal peptide to the N-terminus of the *PvH3.3* gene (Pavir.Ib01857.1) that also contained an N-terminal YFP tag (Figure S3). As shown in Figure 2D, the Myr-YFP-PvH3.3 fluorescence signal was primarily detected on the plasma membrane with no obvious nuclear localization. Interestingly, the fusion of the myristoylation signal peptide could completely inhibit the cell death phenotype triggered by YFP-PvH3.3. This suggests that the PvH3.3 protein needs to be localized in the plant nucleus in order to trigger cell death.

### Overexpression of the N-terminal tail of *PvH3.3* and *PvCENH3* triggers programmed cell death of transformed tobacco cells

**Figure 3:**
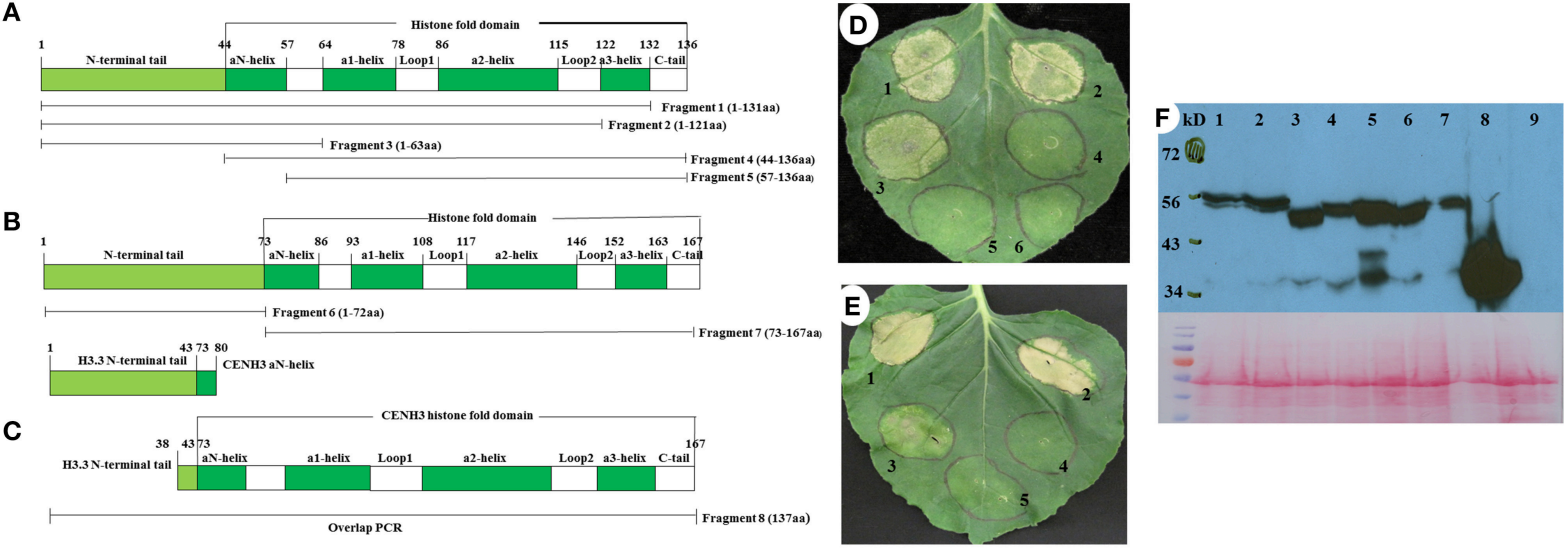
**Diagram of eight fragments of the switchgrass histone H3.3 and CENH3, and the phenotype of overexpression of different fragments of switchgrass ***histone H3.3*** and ***CENH3*** in ***N. benthamiana*****. **(A)** PCR primer location for different fragments of switchgrass histone H3; **(B)** PCR primer location for different fragments of switchgrass CENH3; **(C)** The chimeric gene contains the N-terminal tail of switchgrass histone H3.3 and the histone-fold domain of CENH3 was constructed by using overlap PCR. Agrobacterium strains expressing different DNA fragments as outlined in **(A-C)** were inoculated in *N. benthamiana* and the cell death phenotype were pictured at 4 days post inoculation. **(D)** 1, Fragment 1-YFP; 2, Fragment 2-YFP; 3, Fragment 3-YFP; 4, Fragment 4-YFP; 5, Fragment 5-YFP; 6, YFP only (negative control); **(E)** 1, PvCENH3-YFP; 2, Fragment 8-YFP; 3, Fragment 6-YFP; 4, Fragment 7-YFP; 5, YFP only. **(F)** Western blot to detect switchgrass histone H3-YFP fusion proteins. Different fragments of histone H3-YFP fusion proteins transiently expressed in *N. benthamiana* plant cells were detected by western blot. 1. Fragment 1-YFP; 2. Fragment 2-YFP; 3. Fragment 3-YFP; 4. Fragment 4-YFP; 5. Fragment 5-YFP; 6. Fragment 6-YFP; 7. Fragment7-YFP; 8.YFP only (negative control); 9*. N. benthamiana* total proteins (negative control); the western blot membrane was also stained with Ponceau S Staining Solution to show the equal loading of each protein samples.

Histone H3 proteins have two domains: an N-terminal tail and a histone fold domain (Luger et al., 1997; Malik and Henikoff, 2003). In order to determine the part of the PvH3.3 protein that is essential for triggering cell death in *N. benthamiana*, we performed a deletion mutagenesis series from both the N- and C-terminal ends. Five different fragments of *PvH3.3* (Figure 3A) were used to generate *PvH3.3-YFP* fusion genes. As shown in Figure 3D, the N-terminal tail (1–43aa) is the part of the protein that maintains the ability to trigger cell death. All of the fragments that contained the N-terminal tail were predominately localized in the nucleus (Figures S4A–C). This suggests that there is an unidentified nuclear localization signal in the N-terminal tail sequence. The fragments that contained solely the PvH3.3 C-terminal histone fold domain, however, lost the ability to localize in the nucleus (Figures S4D,E) and could not trigger cell death in the transformed *N. benthamiana* plant cells (Figure 3D).

To investigate whether the N-terminal of *PvCENH3* is also sufficient enough to cause cell death, both the N-terminal tail and the histone fold domain of the *PvCENH3* gene were amplified (Figure 3B) and cloned into expression vectors that fused them with a C-terminal YFP tag. After transiently expressing each construct in tobacco, the fluorescence signal showed that the N-terminal tail of PvCENH3 solely localized in the nucleus, whereas the histone fold domain was ambiguously located between the nucleus and the cytosol (Figures S4F,G). Therefore, PvCENH3 must also contain an uncharacterized nuclear localization signal in its N-terminal tail. Similarly to the results for *PvH3.3*, overexpression of the N-terminal tail caused cell death after 3 dpi, whereas the histone fold domain failed to trigger cell death (Figure 3E). A previous report has suggested that a chimerical histone *H3-CENH3* fusion gene can trigger chromosome elimination in Arabidopsis (Ravi and Chan, 2010). In this study, we also fused the N-terminal tail of *PvH3.3* with the histone fold domain of *PvCENH3* (Figure 3C). We found that transient expression of *PvH3.3*-*PvCENH3*-*YFP* could also trigger cell death in *N. benthamiana* plant cells (Figure 3E). The expressions of all different fragments of *PvH3.3* and *PvCENH3* in *N. benthamiana* were confirmed by Western blot analyses (Figure 3F).

### Silencing of the *SGT1* gene in *N. tabacum* inhibits the cell death phenotype triggered by *PvH3.3* and *PvCENH3*

**Figure 4:**
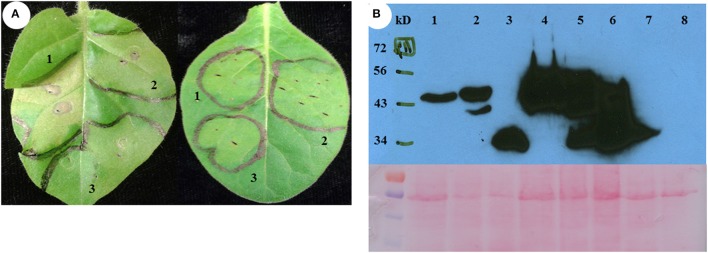
**The phenotype of transient expression of different fragments as outlined in Figure 3 of switchgrass H3 in ***N. tabacum*****. **(A)** Transient assay phenotype. Left is non-transgenic plant, right is *NtSGT1-RNAi* transgenic plant. 1, Fragment 3-YFP; 2, Fragment 6-YFP; 3,YFP only (negative control). **(B)** Different fragments of histone H3-YFP fusion proteins transiently expressed in *NtSGT1-RNAi* transgenic *N. tabacum* and non-transgenic *N. tabacum* plant cells were detected by western blot. 1, Fragment 3-YFP in transgenic plant; 2, Fragment 6-YFP in transgenic plant; 3, YFP only in transgenic plant; 4, Fragment 3-YFP in non-transgenic plant; 5, Fragment 6-YFP in non-transgenic plant; 6, YFP only in non-transgenic plant; 7, *NtSGT1-RNAi* transgenic *N. tabacum* total proteins (negative control); 8, Non-transgenic *N. tabacum* total proteins (negative control).

The cell death phenotype caused by the overexpression of PvH3.3 and PvCENH3 is similar to the hypersensitive response (HR) triggered by the interaction between plant pathogen effectors and cognate plant *R* genes (Coll et al., 2011). Since the HR-like cell death that is triggered by many *R* genes requires the function of SGT1, which is a conserved immune signaling component (Peart et al., 2002), we therefore tested whether or not *SGT1* is also required for the elicitation of cell death induced by overexpression of PvH3.3 and PvCENH3 in *Nicotiana tabacum*. Two independent *NtSGT1*-RNAi transgenic lines (Traore et al., under revision) were used for transient overexpression of PvH3.3 and PvCENH3. As shown in Figure 4A, transient expression of *PvH3.3*^(1−63*aa*)^*-YFP* and Pv*CENH3*^(1−72*aa*)^*-YFP* triggered cell death phenotypes on the wild type *N. tabacum* plants but failed to trigger any phenotype on the *NtSGT1*-RNAi plants. Western blot analysis found that the PvH3.3^(1−63*aa*)^-YFP and PvCENH3^(1−72*aa*)^-YFP fusion proteins were both expressed in the wild type and the *NtSGT1*-RNAi transgenic plants (Figure 4B). Therefore, Nt*SGT1* is essential for promoting histone H3-mediated cell death. Further studies are needed to investigate the role that SGT1 may play in histone gene-mediated cell death.

## Discussion

In this study, we identified 17 potential switchgrass histone *H3* genes in the draft switchgrass genome (v1.1). Although, their nucleotide sequences differ, these 17 genes encode for 9 individual proteins. Despite being a tetraploid, only two of these genes represent putative *CENH3* genes, which is the key histone component of the centromere. RNA-seq analysis of the histone *H3* genes in leaf tissue of Alamo and Dacotah found that some of the histone *H3* genes have sequence polymorphisms at the nucleotide level (Table S2). Alamo and Dacotah represent lowland and upland switchgrass ecotypes, respectively, and are genetically diverse from each other. Therefore, the identified SNPs could be developed into molecular markers that can distinguish histone *H3* gene alleles between the different ecotypes of switchgrass. We also found that transient overexpression of several of the putative switchgrass histone H3 proteins in tobacco leaves can trigger cell death.

### Histone *H3* genes in switchgrass

Plant histone *H3* genes belong to a gene family with multiple members. For instance, maize and rice have approximately 14–15 histone *H3* genes (Ingouff and Berger, 2010). Additionally, Arabidopsis contains 15 histone *H3* genes including five *H3.1* genes, one *H3.1-like* gene, three *H3.3* genes, five *H3.3-like* genes, and one *CENH3* gene (Okada et al., 2005). In this study, we used a homology-based approach to identify histone *H3* genes in switchgrass. We found six *H3.1* genes, three *H3.3* genes, four *H3.3-like* genes, two *H3.1-like* genes, and two *CENH3* genes in the switchgrass genome. Core histones are among the most highly conserved proteins in eukaryotes, emphasizing their important role in maintaining genome stability and structure (Marino-Ramirez et al., 2011). In this study, we were also able to determine that histone H3 proteins from different species are highly conserved. The histone H3 proteins and the CENH3 proteins share a relatively conserved histone fold domain; however, the N-terminal tail of each protein group shows significant sequence divergence, both among species and between each group (Figure 1A). This is consistent with previous studies showing that CENH3 has evolved rapidly, particularly in its N-terminal tail, and the adaptive evolution that has occurred in the N-terminal tail may be in response to changing centromeric satellite repeats (Henikoff et al., 2001; Malik and Henikoff, 2001; Talbert et al., 2002).

In this study, 10 *PvH3* genes were cloned from switchgrass leaf cDNA. The other *PvH3* genes may be expressed at undetectable levels in switchgrass leaves but may be significantly expressed in other tissue types. For example, *AtMGH3*, which is an Arabidopsis histone H3 gene, can only be detected in mature buds and mature bicellular and tricellular pollen (Okada et al., 2005).

### The subcellular localization of histone H3

The fluorescent signals of the histone H3-YFP fusion proteins were mainly located in the nucleus. This is similar to the histone H3 that was studied in *Drosophila* (Ahmad and Henikoff, 2002b). The histone H3 fused with two tandem repeats of GFP in yeast cell is also in the nucleus (Mosammaparast et al., 2002). Using the pSORT software (http://www.psort.org/), we identified a putative nuclear localization signal (NLS), “KRVTIMPKDIQLARRIR,” which is conserved among the switchgrass histone H3 proteins. This NLS is located in the Loop2 and α3-helix of the protein. Two DNA binding motifs, “KAPRKQL” and “PFQRLVREI,” were identified in the N-terminal tail and the α1-helix, respectively (Figure 1A). In our transient assay experiments, however, only the N-terminal tail fused with YFP was predominantly localized in the plant nucleus and the fragments that did not contain the N-terminal tail lost the ability to localize in the nucleus (Figure S4). The result is similar with the H3 and H4 GFP fusion proteins in yeast cells. The amino-terminal domains of yeast H3-(1–58) and H4-(1–42) are necessary and sufficient for nuclear transport. While truncated H3 or H4 proteins that lacked amino terminal H3-(58–136) and H4-(42–103) resulted in reduced nuclear accumulation (Mosammaparast et al., 2002). Previous studies have indicated that the histone-fold domain of CENH3 controls kinetochore localization (Sullivan et al., 1994; Lermontova et al., 2006; Black et al., 2007). In this study, we demonstrated that the N-terminal tail of PvCENH3 is essential and sufficient for its nuclear localization (Figure S4). Interestingly, we identified a putative NLS signal, “PKKKLQF,” in the N-terminal tail of the switchgrass CENH3. Previous studies have reported that NLS activity is found in the N-terminal tail domains of all core histones (Baake et al., 2001; Mosammaparast et al., 2001). Therefore, switchgrass histone H3 maybe also contains a NLS at its N-terminal tail. Site-directed mutagenesis on this putative NLS signal will be necessary in order to further validate its function.

### Overexpression of histone *H3* triggers cell death in *N. benthamiana* plant cells

In this study, we found that overexpression of switchgrass histone *H3* genes could trigger a cell death phenotype in *N. benthamiana*. Since overexpression of histone *H3* genes of *N. benthamiana*, Arabidopsis and Italian ryegrass also triggered a similar cell death, the cell death phenotype that we observed is not simply due to the heterologous expression of the switchgrass histone *H3* genes in *N. benthamiana*. Therefore, aberrant expression of histone *H3* genes appears to be toxic to plant cells. The cytotoxicity of histones has been previously reported in yeast and mammalian cells (Singh et al., 2010). A delicate balance between histone protein concentrations and DNA synthesis during the packaging of the genome into chromatin is essential for cell viability (Singh et al., 2010). Insufficient amounts of histone proteins inside the cell have been shown to be lethal (Han et al., 1987). On the other hand, excessive levels of histone proteins have also proved to be deleterious for cell growth as they promote genomic instability, increase DNA damage sensitivity and accelerate cytotoxicity (Gunjan and Verreault, 2003; Singh et al., 2009). The toxicity of large amounts of histone proteins could be attributed to their highly positive charge, which may exhibit non-specific electrostatic interactions with many negatively charged subcellular molecules including nucleic acids, such as DNA and RNA, and negatively charged proteins (Singh et al., 2010). Interestingly, mammalian histones have been found in the extracellular space between cells. These extracellular histones may bind to cell membrane receptors and may activate multiple signaling pathways that can trigger diverse cellular responses including cytotoxicity, proinflammation, procoagulation and barrier dysfunction (Chen et al., 2014; Xu et al., 2015).

Chronic obstructive pulmonary disease (COPD) is a progressive disease that is characterized by extensive lung inflammation and apoptosis of pulmonary cells. Inflamed lung cells trigger an 8-fold increase in production of hyperacetylated histone H3.3, a modified version of this histone protein that is resistant to proteasomal degradation. As a result, the damaged cells release acetylated H3.3 into the extracellular space where it binds to lung structural cells and induces apoptosis (Barrero et al., 2013). Xu et al. found that a mixture of histones was cytotoxic to the endothelial cells (EA.hy926) of sepsis patients, and that the toxic effects were mainly due to histones H3 and H4 (Xu et al., 2009). In addition, sera from patients with sepsis directly induced histone-mediated cardiomyocyte death *ex vivo* (Alhamdi et al., 2015). *In vivo* studies on septic mice also confirmed the cause-effect relationship between circulating histones and the development of cardiac injury, arrhythmias and left ventricular dysfunction (Alhamdi et al., 2015). Therefore, the over-production of histones, either inside of cells or in the extracellular space, is toxic in mammalian cells. In the future, it will be interesting to test if increased levels of extracellular histones can also trigger cell death in plants.

The cytotoxicity caused by aberrant levels of histone proteins is normally avoided by regulating histone gene expression (Gunjan and Verreault, 2003). Correspondingly, other studies have also suggested that histone *H3* gene expression is tightly regulated, either at the transcriptional or translational levels, in different tissue types and during various developmental stages (Reichheld et al., 1998; Marino-Ramirez et al., 2005; Forcob et al., 2014). Further study on the regulation mechanisms underlying histone *H3* gene expression may help us gain a deeper understanding of the biological functions of histone H3 proteins.

The cell death phenotype observed in this study is similar to the hypersensitive response (HR) triggered by the interaction between plant disease resistance (R) proteins and their cognate effectors (Coll et al., 2011). The HR-like cell death is usually associated with ion fluxes across the plasma membrane and a burst of reactive oxygen species, such as H_2_O_2_ and superoxide anion radicals. This leads to increased cytosolic Ca^2+^ levels, activated protein kinase cascades, global transcriptional reprogramming, nuclear DNA cleavage, rapid cytoskeletal reorganization, organelle dismantling and eventually results in a rapid cell death (Pontier et al., 1998). In the future, it will be interesting to investigate if the cell death triggered by histone *H3* genes activates signaling cascades that overlap with the HR-like cell death triggered by R proteins. In this study, we demonstrated that silencing of *SGT1*, which promotes HR-like cell death in response to plant pathogens, in *N. tabacum* could completely abolish the cell death phenotype triggered by PvH3s (Figure 4A). Therefore, SGT1 may also function in promoting histone H3-mediated cell death.

A previous study in mice has suggested that CENH3 fused with GFP may have altered protein function and thus may affect mice embryo development (Kalitsis et al., 2003). For example, transgenic mice carrying the heterozygous *CENPA-GFP/CENPA* alleles were healthy, fertile and normal, whereas the mice carrying homozygous *CENPA-GFP/CENPA-GFP* alleles had delayed development and died during the embryo development stage (Kalitsis et al., 2003). It is possible the homozygous transgenic mice have increased CENPA-GFP protein accumulation that is lethal. We speculate that the GFP fused to switchgrass CENH3 may also have resulted in a high accumulation of PvCENH3-GFP proteins and causing cell death phenotype in the transient assay on *N. benthamiana* plants. However, further investigation is needed to understand the mechanism of cell death caused by CENPA-GFP in either mice or *N. benthamiana*.

Recent studies have shown that transgenic Arabidopsis plants expressing a chimeric GFP-tailswap protein (N-terminal tail of CENH3 was replaced by H3.3 N-terminal tail) display abnormal chromosome segregation during meiosis (Ravi and Chan, 2010; Lermontova et al., 2011). When the transgenic Arabidopsis carrying GFP-tailswap were crossed with a wild type plant, the GFP-tailswap derived genome was eliminated from the zygotes of some F_1_ individuals, thus generating a high proportion of haploid (45%) and aneuploid (28%) progenies (Ravi and Chan, 2010). In this study, we also generated a chimerical switchgrass *CENH3* gene by replacing the N-terminal tail of *CENH3* with the *PvH3.3* N-terminal tail. Our transient assay results showed that this chimerical gene was also able to trigger a cell death phenotype (Figure 3E). Therefore, we speculate that the Arabidopsis chimeric GFP-tailswap gene may have altered native gene expression levels or resulted in histone protein accumulation in transgenic Arabidopsis, which ultimately was lethal to the plant cells. Further studies are needed to fully investigate the mechanisms underlying GFP-tailswap toxicity in Arabidopsis.

In this study, we revealed that the overexpression of the N-terminal tail of histone *PvH3.3* in the nucleus is essential and sufficient for triggering a cell death phenotype. Histone H3 proteins are important components of the nucleosome and play critical roles in the formation of higher-order chromatin (Dorigo et al., 2004; Kan et al., 2007; Sperling and Grunstein, 2009). Previous studies have demonstrated that histone N-terminal tails play an important role in the structure and stability of nucleosomes. The N-terminal truncation of histone H3 and CENH3 proteins enhances the transient unwrapping of DNA at the ribosomal entry/exit regions, which disrupts some histone-DNA contacts and thus reduces chromatin stability (Biswas et al., 2011; Tachiwana et al., 2011; Iwasaki et al., 2013). We speculate that overexpression of the histone *H3* N-terminal tail may compete with native histone H3s to interact with the nucleosomes, thus causing chromosomal instability. This interference may also lead to aberrant gene expression and disrupt faithful DNA replication, ultimately resulting in a cell death phenotype. In the future, identification of genes that are either up- or down-regulated, through CHIP-seq or RNA-seq, by the overexpression of histone *H3* may allow us to understand how these proteins can contribute to cell death.

## Accession numbers

The RNA-seq data used in this article can be found in the GenBank database under the following accession numbers: SRR3473343, SRR3473344, SRR3467193, SRR3467194, SRR3467195, SRR3467196, SRR3467197, and SRR3467198.

## Author contributions

BZ, XZ designed the research projects. JM, TF performed the experiments. JM, TF, LH, XZ, and BZ wrote the manuscript. All authors reviewed and edited the manuscript before submission.

### Conflict of interest statement

The authors declare that the research was conducted in the absence of any commercial or financial relationships that could be construed as a potential conflict of interest.

## Supplementary material

Figure S1 **Switchgrass histone H3.3 variant exons and introns alignment**. The position of three introns were highlighted with black line on the top of the DNA sequence. The conserved nucleotides are highlighted in red. The polymorphic nucleotides A and G are in blue, while C and T are in black.

Figure S2 **Subcellular localization of different histone H3-YFP fusion proteins. (A)** PvH3.3-YFP predominately localized into plant nucleus of the transformed tobacco plant cells; **(B)** PvCENH3-YFP predominately localized into plant nucleus of the transformed tobacco plant cells; **(C)** YFP only (negative control) localized in both cystosol and nucleus of the transformed tobacco plant cells.

Figure S3 **The diagram of binary vector pEG203-MyrYFP for expressing proteins fused with an N-terminal myristoylation signal peptide**. A DNA fragment contains an N-terminal myristoylation signal peptide fused with YFP gene was amplified using overlap PCR method. The derived fragment was inserted into pEarleyGate 203 to generate pEG203-MyrYFP. Myr, Myristoylation signal peptide; TEV, TEV protease cleavage site.

Figure S4 **Localization of different fragments outlined in Figure 3 of H3.3-YFP and CENH3-YFP fusion proteins in the transformed tobacco plant cells. (A)** Fragment 1-YFP; **(B)** Fragment 2-YFP; **(C)** Fragment 3-YFP; **(D)** Fragment 4-YFP; **(E)** Fragment 5-YFP; **(F)** Fragment 6-YFP; **(G)** Fragment 7-YFP.

Table S1 **Primer sequences used in this study**.

Table S2 **SNPs identified between cv. Alamo and cv. Dacotah. SNV, single nucleotide variant**.
