# Supplementary material for: Identification and Characterization of Switchgrass Histone H3 and CENH3 Genes
Source: Front Plant Sci. 2016 Jul 12;7:979. doi: 10.3389/fpls.2016.00979 (PMC4940616; doi:10.3389/fpls.2016.00979)
Supplement: Figure S1 — Switchgrass histone H3.3 variant exons and introns alignment. The position of three introns were highlighted with black line on the top of the DNA sequence. The conserved nucleotides are highlighted in red. The polymorphic nucleotides A and G are in blue, while C and T are in black. [file Image1.pdf]

```
Pavir.Ia031212   1 ATGGCCCGTACCAAGCAAACTGCTCGCAAGTCCACGGGAGGGAAGGCTCCCAGGAAGCAG
Pavir.Ib018571   1 ATGGCCCGTACCAAGCAAACCGCTCGCAAGTCCACGGGAGGGAAGGCTCCCAGGAAGCAG
Pavir.J05563.1   1 ATGGCCCGTACGAAGCAGACCGCCCGCAAGTCCACCGGAGGGAAGGCCCCCCGCAAGCAG
Pavir.J26857.1   1 ATGGCTCGTACTAAGCAGACCGCTCGCAAGTCCACTGGAGGCAAGGCTCCTAGGAAGCAA
Pavir.J09299.1   1 ATGGCTCGTACTAAGCAAACCGCTCGCAAGTCCACTGGAGGAAAGGCTCCTAGGAAGCAA
Pavir.J24812.1   1 ATGGCTCGTACTAAGCAGACCGCTCGCAAGTCCACTGGAGGAAAGGCTCCTAGGAAGCAA
Pavir.J10481.1   1 ATGGCTCGCACTAAGCTGACGGCTCGCAAGTCAACCGGAGGAAAGGTCCCCAGGAAGCAA

Pavir.Ia031212  61 CTTGCAACTAAGGTAAGG--------TTC-----------------TTCATATATGCTTT
Pavir.Ib018571  61 CTTGCAACTAAGGTAAGG--------TTC-----------------TTTATATATTCTTT
Pavir.J05563.1  61 CTCGCCACCAAGGTTGGT--------TCTCGTGCTC-----TTCCGTTCCGATCTGCTCT
Pavir.J26857.1  61 CTAGCCACCAAGGTTTGTATATTTATTCTTGTGCCT--TTTTTTTGTTACTGTCTTCAAC
Pavir.J09299.1  61 CTAGCCACCAAGGTTTGTAAATTTATTCTTATGCCA--TTTTTTTGTTACTGTCTTCAAC
Pavir.J24812.1  61 CTAGCCACCAAGGTTTGTATATTCAGTCTTATGCCAATCTTTTTTGTTACTATGTTCATT
Pavir.J10481.1  61 CTCGTTGCCAAG------------------------------------------------

Pavir.Ia031212  96 TTTGTTGTTAGA-------------------------------------CGTTGATA--T
Pavir.Ib018571  96 CTTGTTGCTCGG-------------------------------------CATTGATA--T
Pavir.J05563.1 108 GTTGTCGCCGAG-------------------------------------ATTTGGCT--G
Pavir.J26857.1 119 TGTGCTGCCCAATGACTTTACTTTATCATTGATATGGGAACAAAC--ATCTCTGGCTACT
Pavir.J09299.1 119 TATGCTGCCCAA-------------------------------------CTCTGGCTACT
Pavir.J24812.1 121 AATGCTGCACAATGACTTTACTTTATCATTGATATGGAAAGAAAATGATCTTTGGCTACT
Pavir.J10481.1  72 ------------------------------------------------------------

Pavir.Ia031212 117 TGGTTTGAAGTT-----------------TCAACTTAA----------TGA----AACCC
Pavir.Ib018571 117 TGGTTTGAAGCC-----------------TCAACTTAA----------TGA----AACTC
Pavir.J05563.1 129 CGGTTGTTTGGG-----------------CCGAGTTGTCC--------TGA----GACGC
Pavir.J26857.1 177 TTGTTTTAGGGTGAAAAAGTTATCTGTTGTCTAATTATAGACGACATCTGACTTCAACGC
Pavir.J09299.1 142 CTGTTTTAGGGTGGAAAAGTTATCTGTTGTCTACTTATAG---ATATCTGACTTCAATGC
Pavir.J24812.1 181 CTGTTTTAGGGT--------TCCCTGTTGGATAATTATAG---AAATCTGAATTTAATGC
Pavir.J10481.1  72 ------------------------------------------------------------

Pavir.Ia031212 146 -------------------TTTGTAA---------------------TA--TTGCTTCAT
Pavir.Ib018571 146 -------------------TTTGTAA---------------------T---TTGCTTCAT
Pavir.J05563.1 160 G------------------TTTGCGA---------------------TTCGCTGCTGTGC
Pavir.J26857.1 237 ATAGGCTCAATTAGTTTTATTTGCAGAATGGTTATTTACTATGTGACTTTGTTCTTTGAC
Pavir.J09299.1 199 GTTGGTTCTATTAGTTTTATTTGCAAAATGGTTATTTACTATGTGACTTTGTTTTTTGAC
Pavir.J24812.1 230 ATTCGTTCTGTTAGTTTTATTTGCAAAATGGTTATTTACTATGTGACTTTGTTCTTTGAC
Pavir.J10481.1  72 ------------------------------------------------------------

Pavir.Ia031212 164 AGGCTGCCCGTAAGTCAGCCCCGACCACTGGAGGAGTGAAGAAGCCCCATCGCTACCGCC
Pavir.Ib018571 163 AGGCTGCCCGCAAGTCAGCCCCGACCACTGGAGGAGTGAAGAAGCCCCACCGCTACCGCC
Pavir.J05563.1 181 AGGCGGCGAGGAAGTCGGCGCCGACGACCGGCGGCGTGAAGAAGCCCCACCGCTACAGGC
Pavir.J26857.1 297 AGGCTGCCCGTAAGTCTGCGCCCACAACTGGGGGAGTGAAGAAGCCTCACCGTTACCGCC
Pavir.J09299.1 259 AGGCTGCCCGTAAGTCTGCGCCCACAACTGGGGGAGTGAAGAAGCCTCACCGTTACCGCC
Pavir.J24812.1 290 AGGCTGCCCGTAAGTCTGCGCCCACAACTGGGGGAGTGAAGAAGCCTCACCGTTACCGCC
Pavir.J10481.1  73 --GATGCCCGTAAGTCAGCTCCGACAACCGGAGGAGTCAAGAAGCCTCGCCGTTACCGCC

Pavir.Ia031212 224 CTGGCACTGTTGCCCTCCGGTTAGTA----------AAATT-------------T-----
Pavir.Ib018571 223 CTGGGACTGTTGCCCTCCGGTTAGTA----------AAATAATA----------T-----
Pavir.J05563.1 241 CCGGGACCGTGGCGCTCCG-----------------------------------------
Pavir.J26857.1 357 CTGGGACTGTTGCTCTTCGGTGTGTG----------AAATACTTAAATGTTATTC-----
Pavir.J09299.1 319 CTGGGATTGTTGCTCTTCGGTGTGTG----------AAATACTGAAATATTATTC-----
Pavir.J24812.1 350 CTGGAACTGTTGCTCTTCGGTGTGTG----------AAATACTAAAATATTATTC-----
Pavir.J10481.1 131 CTGGAACGGTAGCTCTTCGGTATGTATGTGCAGCACAGCTGATTTCATTTCACTTCCGGT

Pavir.Ia031212 256 -CCTTGAATGTTTCTTATGTCCAGCCTAGT-----------GTTTTGTTGTTTGATTGAT
Pavir.Ib018571 258 -CCTTGAATGTTTCT-ATGTCCAGCCTAGT-----------ATCCTGCTGCTTGATTGAT
Pavir.J05563.1 259 ------------------------------------------------------------
Pavir.J26857.1 402 -ATTAGACTTGCTATTGTGCTTACTGAAAT-----------GATTCTCTACTAAATTGGC
Pavir.J09299.1 364 -ATTTGGTTTACTATTGTACTAACTGAAAT-----------GATTCTCTGCCAAATTGGC
Pavir.J24812.1 395 -ATTTGACTTAGTATTGTGCTTACTGAAAT-----------GATTTTCTGCCAAATTGGC
Pavir.J10481.1 191 GATTATGATTTCTATTTCATGAAATAAAATTACCATGCTAGAATTTGACCTCAACTTCAT
```

```
Pavir.Ia031212  304 TTTGCTGTGTCTTGAATGCAA---------------------------------------
Pavir.Ib018571  305 TC-GCTGTGTCTTGAATACAA---------------------------------------
Pavir.J05563.1  259 ------------------------------------------------------------
Pavir.J26857.1  450 AAATTCTAAACTTATGCCTGA---------------------------------------
Pavir.J09299.1  412 AATTTCTAAACTTATGCCTGA---------------------------------------
Pavir.J24812.1  443 AAATTCTCAATTTATTCATGA---------------------------------------
Pavir.J10481.1  251 TCTTATTTTCTTTTTATCTGCAAATTGAATGAACCACTTTAATTTCCTCCCTCCACTTGT

Pavir.Ia031212  325 ---TTTGATAGTGAGATCCGCAAGTACCAGAAGAGCACTGAGCTGTTGATCAGGAAGCTG
Pavir.Ib018571  324 ---TTTGATAGTGAGATCCGCAAGTACCAGAAGAGCACTGAGCTGTTGATCAGGAAGCTG
Pavir.J05563.1  260 -----------CGAGATCCGCAAGTACCAGAAGAGCACGGAGCTCCTGATCCGCAAGCTC
Pavir.J26857.1  471 ---TTGTACAGTGAAATCCGCAAGTACCAGAAGAGCACTGACCTGCTCATAAGGAAGCTT
Pavir.J09299.1  433 ---TTCTGCAGTGAAATCTGCAAGTACCAGAAGAGCACTGACCTGCTCATAAGGAAGCTT
Pavir.J24812.1  464 ---TTCTGCAGTGAAATCCGCAAGTACCAGAAGAGCACTGACCTGCTCATTAGGAAGCTT
Pavir.J10481.1  311 TCCTCCTCCAGTGAAATTCGCAAGTACCAAAAGGGCGCCGAGCTGCTCATTAGGAAGATG

Pavir.Ia031212  382 CCCTTCCAGAGGCTTGTCAGGGAAATTGCACAGGACTTCAAGGTTT----GATATCTCTG
Pavir.Ib018571  381 CCCTTCCAGAGGCTTGTTAGGGAAATTGCACAGGACTTCAAGGTCA----GATATCTATG
Pavir.J05563.1  309 CCCTTCCAGCGCCTGGTCCGCGAGATCGCGCAGGACTTCAAG------------------
Pavir.J26857.1  528 CCATTCCAGAGGCTTGTTAGGGAGATTGCCCAGGATTTCAAGGTGA----GATTTGTATT
Pavir.J09299.1  490 CCATTCCAGAGGCTTGTTAGGGAGATTGCCCAGGATTTCAAGGTGA----GATTTGTATT
Pavir.J24812.1  521 CCATTCCAGAGGCTTGTTAGGGAGATTGCCCAGGATTTCAAGGTGA----GATTTGTATT
Pavir.J10481.1  371 CCCTTCCAGAGGCTTGTCAGGGAGATTGCCCAGTTCCACAAGGTAATTTAAATATATGTT

Pavir.Ia031212  438 AATTGAACTTGTATAT---------------GA--GATTGTGTTTTGGCACTTTATC---
Pavir.Ib018571  437 AATTGAAC--AAATAT---------------GA--GATTCTGTCTTGGCACTTTATC---
Pavir.J05563.1  350 ------------------------------------------------------------
Pavir.J26857.1  584 CATACTGTTGTATTGT---------------GATCAGTCATGTGATGCCACAGTTCTAAA
Pavir.J09299.1  546 CATATTGCTATATTGT---------------GATCAGTCATGTGGTGCCACAGTTCT---
Pavir.J24812.1  577 CAAACTGCTAAATTGT---------------GATCAGTCATGAGATGCCACAGTTCT---
Pavir.J10481.1  431 TGGTTTGTCTTGATATTTGAAACATCTGTAGGATCATCTAAATACTAATAGCATTTTTTT

Pavir.Ia031212  478 ---------TGGTTCTTGATTTGTGGTTGATCAAACCCG-TTCTTGTTTTGCAGACTGAT
Pavir.Ib018571  475 ---------TGTTTCTTGATGAGTGGTTGATCAAACTCGGTTCTTGTTTTGCAGACTGAT
Pavir.J05563.1  351 ------------------------------------------------------ACGGAC
Pavir.J26857.1  629 GTAGTGAAGTATAACTAGAAACTTTTCTTGACCTCTTGCTGTGTATTTGTGCAGACTGAT
Pavir.J09299.1  588 -----GAAGTATAACTAGAAACTTCCCTTAACCTCTTCCTGTGTATTTGTGCAGACTGAT
Pavir.J24812.1  619 -----GAAGTATA--------------TTAACCTCTTGCTGTGTATTTGTGCAGACTGAT
Pavir.J10481.1  491 TCCATGGAGTTCTTATATATGTACTGTTCCATCACTTGATGGCTTTGAATGCAGAGTGAC

Pavir.Ia031212  528 CTGCGTTTCCAGAGCCATGCTGTGCTTGCCCTCCAGGAGGCTGCCGAGGCGTACCTTGTT
Pavir.Ib018571  526 CTGCGTTTCCAGAGCCATGCTGTGCTTGCCCTCCAGGAGGCTGCCGAGGCGTACCTTGTT
Pavir.J05563.1  357 CTCCGGTTCCAGAGCCACGCCGTGCTGGCGCTCCAGGAGGCCGCCGAGGCCTACCTCGTC
Pavir.J26857.1  689 CTGCGTTTCCAGAGCCATGCGGTGCTTGCCCTGCAGGAGGCTGCGGAAGCATACCTGGTG
Pavir.J09299.1  643 CTGCGTTTCCAGAGCCATGCGGTGCTTGCCCTGCAGGAGGCTGCGGAAGCATACCTGGTG
Pavir.J24812.1  660 CTGCGTTTCCAGAGCCATGCGGTGCTTGCCCTGCAGGAGGCTGCGGAAGCATACCTGGTG
Pavir.J10481.1  551 TTGCGCTTCCAGAGCCATGCAGTGCTTGCTTTGCAGGAGGCAGCAGAAGCATACCTCGTG

Pavir.Ia031212  588 GGTCTGTTTGAGGACACCAACCTGTGCGCCATCCATGCTAAGCGTGTGACCATCATGCCT
Pavir.Ib018571  586 GGTCTGTTTGAGGACACCAACCTGTGCGCCATCCATGCTAAGCGTGTGACCATCATGCCC
Pavir.J05563.1  417 GGGCTCTTCGAGGACACCAACCTCTGCGCCATCCACGCCAAGCGCGTCACCATCATGCCC
Pavir.J26857.1  749 GGTCTGTTCGAGGACACCAACCTGTGCGCCATCCACGCCAAGCGTGTGACGATCATGCCT
Pavir.J09299.1  703 GGTCTGTTCGAGGACACCAACCTGTGTGCCATCCACGCCAAGCGCGTGACGATCATGCCC
Pavir.J24812.1  720 GGTCTGTTCGAGGACACCAACCTGTGTGCCATCCACGCCAAGCGCGTGACGATCATGCCC
Pavir.J10481.1  611 GGGCTCTTTGAGGACACCAACTTATGCGCCATCCATGCCAAGCGGGTGACGATCATGCCC

Pavir.Ia031212  648 AAGGACATCCAGCTGGCCAGGAGGATCCGTGGCGAGAGGGCT
Pavir.Ib018571  646 AAGGACATTCAGCTGGCCAGGAGGATCCGTGGCGAGAGGGCT
Pavir.J05563.1  477 AAGGACATCCAGCTCGCCCGCCGCATCCGCGGCGAGCGCGCC
Pavir.J26857.1  809 AAGGACATTCAGCTGGCTAGGAGGATTCGCGGCGAGAGGGCT
Pavir.J09299.1  763 AAGGACATTCAGCTGGCTAGGAGGATTCGCGGCGAGAGGGCT
Pavir.J24812.1  780 AAGGACATTCAGCTGGCTAGGAGGATTCGCGGCGAGAGGGCT
Pavir.J10481.1  671 AAAGACGTTCAGCTGGCTAGGAGGATCCGTGGCGAGAGGACT
```
