# Supplementary figures and images for: Identification and Characterization of Switchgrass Histone H3 and CENH3 Genes
Source: Front Plant Sci. 2016 Jul 12;7:979. doi: 10.3389/fpls.2016.00979 (PMC4940616; doi:10.3389/fpls.2016.00979)

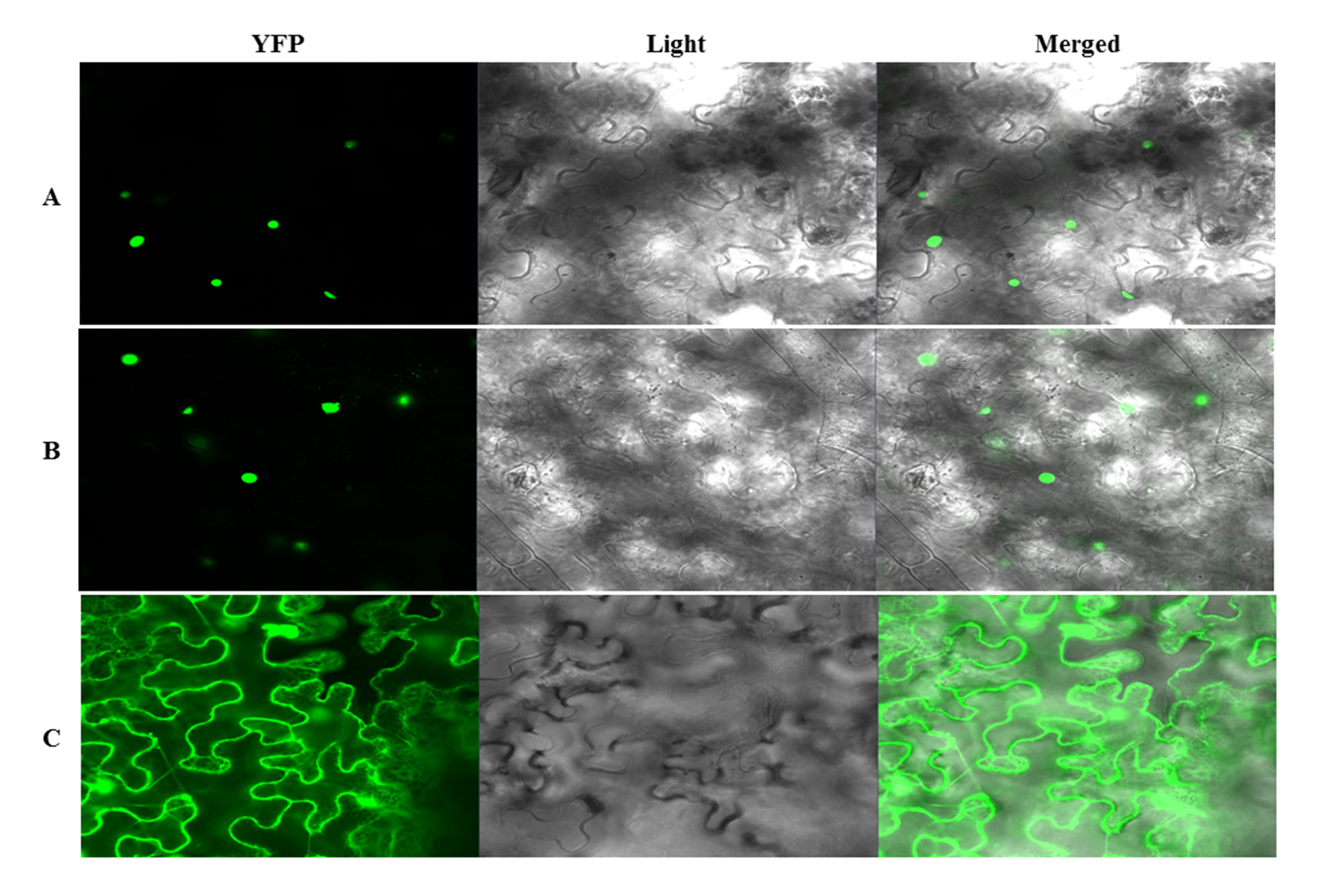

Supplement: Figure S2 — Subcellular localization of different histone H3-YFP fusion proteins. (A) PvH3.3-YFP predominately localized into plant nucleus of the transformed tobacco plant cells; (B) PvCENH3-YFP predominately localized into plant nucleus of the transformed tobacco plant cells; (C) YFP only (negative control) localized in both cystosol and nucleus of the transformed tobacco plant cells. [file Image2.tif]

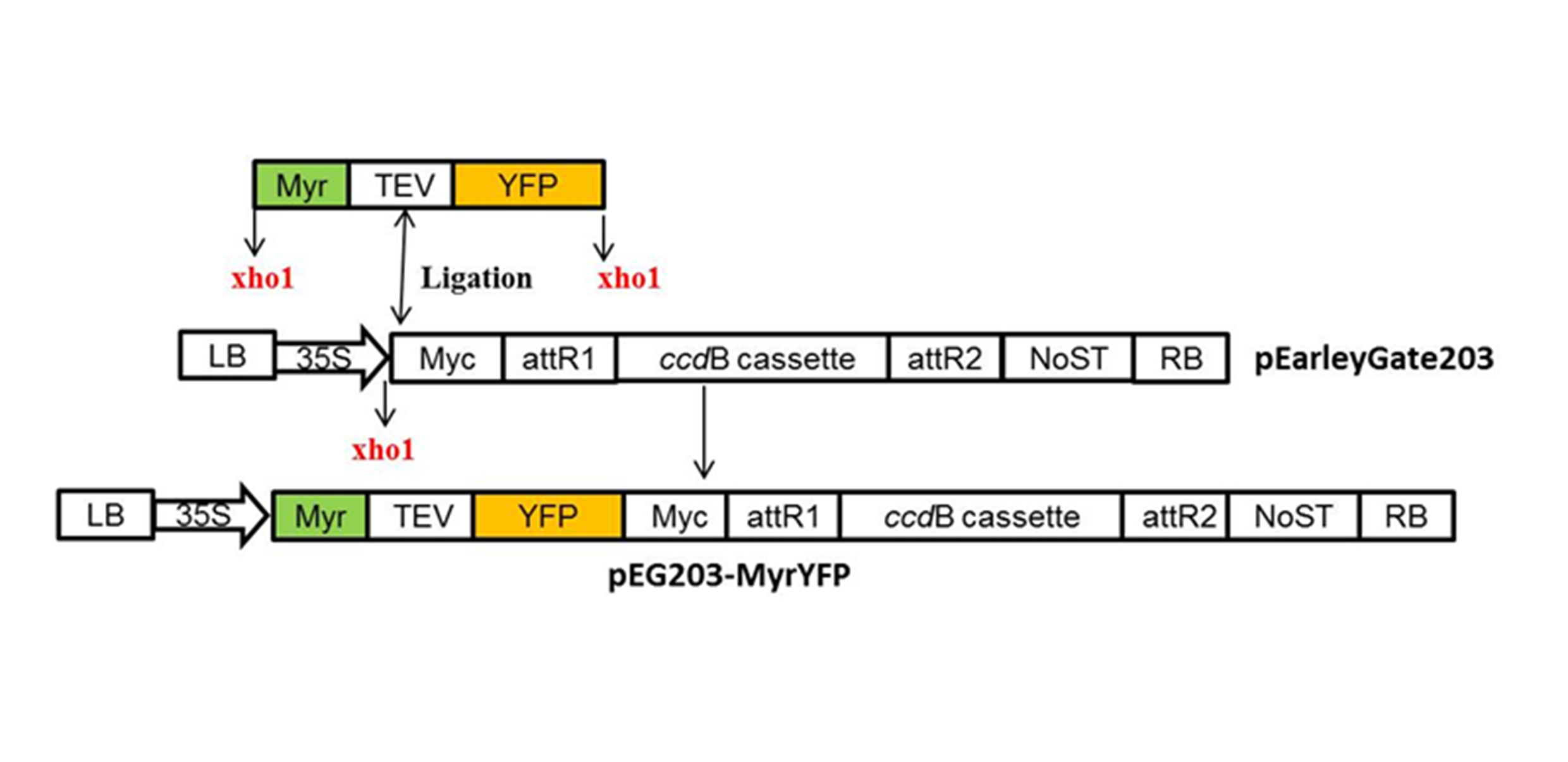

Supplement: Figure S3 — The diagram of binary vector pEG203-MyrYFP for expressing proteins fused with an N-terminal myristoylation signal peptide. A DNA fragment contains an N-terminal myristoylation signal peptide fused with YFP gene was amplified using overlap PCR method. The derived fragment was inserted into pEarleyGate 203 to generate pEG203-MyrYFP. Myr, Myristoylation signal peptide; TEV, TEV protease cleavage site. [file Image3.tif]

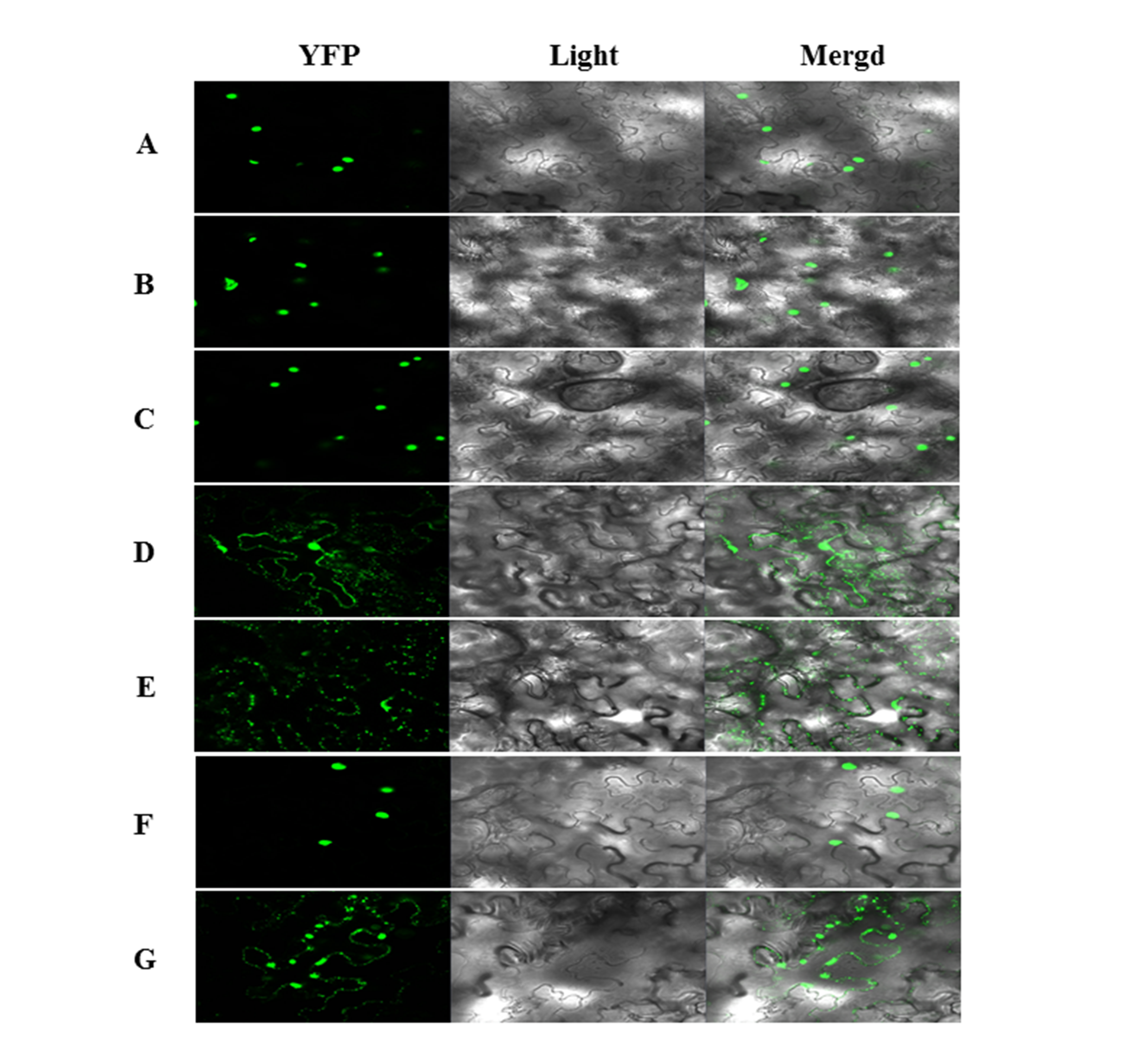

Supplement: Figure S4 — Localization of different fragments outlined in Figure 3 of H3.3-YFP and CENH3-YFP fusion proteins in the transformed tobacco plant cells. (A) Fragment 1-YFP; (B) Fragment 2-YFP; (C) Fragment 3-YFP; (D) Fragment 4-YFP; (E) Fragment 5-YFP; (F) Fragment 6-YFP; (G) Fragment 7-YFP. [file Image4.tif]
