# Supplementary material for: Identification and Characterization of Switchgrass Histone H3 and CENH3 Genes
Source: Front Plant Sci. 2016 Jul 12;7:979. doi: 10.3389/fpls.2016.00979 (PMC4940616; doi:10.3389/fpls.2016.00979)
Supplement: Table S1 — Primer sequences used in this study. [file Table1.docx]

**Table S1 Primer sequences used in this study**

| **Primer name** | | | **Sequence(5′→3′)** |
| --- | --- | --- | --- |
| Conserved  PvH3 | Forward | | caccATGGTGAAGAAGCCCCACCG |
|  | Reverse | | GTGACGCGCTTGGCGTGGATGGC |
| PvH3  primer 1 | Forward | | caccATGGCCCGCACGAAGCAGACGGC |
|  | Reverse | | GGCCCTCTCGCCCCTGATGCG |
| PvH3  primer 2 | Forward | | caccATGGCTCGTACTAAGCAGACCGCT |
|  | Reverse | | AGCCCTCTCGCCGCGAATCCTCCTA |
| PvH3  primer 3 | Forward | | caccATGGCCCGTACGAAGCAGACCGCC |
|  | Reverse | | GGCGCGCTCGCCGCGGATGCGGCGG |
| PvCENH3 | Forward | | caccATGGCTCGCACCAAGCACCC |
|  | Reverse | | CCAGCGCCTTCCACCGATA |
| AtH3 | Forward | | caccATGGCTCGTACGAAGCAAT |
|  | Reverse | | AGCACGCTCTGCACGAATC |
| AtCENH3 | Forward | | caccATGGCGAGAACCAAGCATC |
|  | Reverse | | CCATGGTCTGCCTTTTCCT |
| NbH3 | Forward | | caccATGGCTCGTACCAAGCAAA |
|  | Reverse | | AGCTCGTTCTCCCCTAAT |
| NbCENH3 | Forward | | caccATGGCGAGAACCAAACACCT |
|  | Reverse | | CCAAGGTCGTGCTTTTCC |
| LmH3 | Forward | | caccATGGCCCGTACCAAGCA |
|  | Reverse | | GGCGCGCTCACCGCGGAT |
| Fragment 1 | Forward | | caccATGGCTCGTACTAAGCAGAC |
|  | Reverse | | GCGAATCCTCCTAGCCAGC |
| Fragment 2 | Forward | | caccATGGCTCGTACTAAGCAGAC |
|  | Reverse | | GGGCATGATGGTCACACGC |
| Fragment 3 | Forward | | caccATGGCTCGTACTAAGCAGAC |
|  | Reverse | | CCTGATCAACAGCTCAGTG |
| Fragment 4 | Forward | | caccATGCCTGGGACTGTTGCCCTCC |
|  | Reverse | | AGCCCTCTCGCCGCGAATC |
| Fragment 5 | Forward | | caccATGACTGAGCTGTTGATCAGGA |
|  | Reverse | | AGCCCTCTCGCCGCGAATC |
| Fragment 6 | Forward | | caccATGGCTCGCACCAAGCAC |
|  | Reverse | | TGGCCTCCAACGGTGCGG |
| Fragment 7 | Forward | | caccATGGGGACTGTAGCGCTGCG |
|  | Reverse | | CCAGCGCCTTCCACCGATA |
| Fragment 8 | H3.3  N terminal | Forward | caccATGGCTCGTACTAAGCAGAC |
|  |  | Reverse | TCCCGCAGCGCTACAGTCCCAGGGCGGTAGCGGTGGGGCT |
|  | CENH3 fold-domain | Forward | AGCCCCACCGCTACCGCCCTGGGACTGTAGCGCTGCGGGA |
|  |  | Reverse | CCAGCGCCTTCCACCGATA |
| M13 Forward | | | GTAAAACGACGGCCAG |
| 35S Forward | | | AAGAAGACGTTCCAACCACGTC |
| YFP Forward | | | GACAACCACTACCTGAGCTACC |

Pv: *Panicum virgatum*; At: *Arabidopsis thaliana*; Nb: *Nicotiana benthamiana*; Lm: *Lolium multifolorum*
