# Supplementary material for: Identification and Characterization of Switchgrass Histone H3 and CENH3 Genes
Source: Front Plant Sci. 2016 Jul 12;7:979. doi: 10.3389/fpls.2016.00979 (PMC4940616; doi:10.3389/fpls.2016.00979)
Supplement: Table S2 — SNPs identified between cv. Alamo and cv. Dacotah. SNV, single nucleotide variant. [file Table2.docx]

|  | | | | |  | |  | |  |  |  |  |  |  |  |
| --- | --- | --- | --- | --- | --- | --- | --- | --- | --- | --- | --- | --- | --- | --- | --- |
| **Table S2. SNPs identified between cv. Alamo and cv. Dacotah.**  SNV: single nucleotide variant. | | | | | | | | | | | | | | | |
| **Gene Name** | **Chromosome Location** | **Variant**  **location** | **Variant Type** | **Alamo Variant** | | **Dacotah Variant** | | **Found**  **in AP13** | | **Variant Length** | **Zygosity** | **Read**  **Count** | **Read Coverage** | **Frequency** | **Average**  **base quality** |
| Pavir.Ia03121.2 | Chr09a | 63609261 | Deletion | G | | - | | No | | 1 | Heterozygous | 844 | 1389 | 60.76 | 39.28 |
| Pavir.Ia03121.2 | Chr09a | 63609279 | SNV | T | | A | | No | | 1 | Heterozygous | 509 | 1187 | 42.88 | 39.06 |
| Pavir.Ia03121.2 | Chr09a | 63609131 | SNV | A | | G | | No | | 1 | Homozygous | 112 | 119 | 94.12 | 38.58 |
| Pavir.Ia03121.2 | Chr09a | 63609654 | SNV | C | | A | | No | | 1 | Heterozygous | 5 | 10 | 50.00 | 39.60 |
| Pavir.Ia03121.2 | Chr09a | 63609892 | SNV | C | | T | | No | | 1 | Heterozygous | 16 | 38 | 42.11 | 38.75 |
| Pavir.Ia03121.2 | Chr09a | 63610279 | SNV | T | | A | | No | | 1 | Homozygous | 32 | 33 | 96.97 | 39.75 |
| Pavir.Ib01857.1 | Chr09b | 20162121 | SNV | G | | T | | No | | 1 | Heterozygous | 4806 | 8050 | 59.70 | 38.50 |
| Pavir.Ib01857.1 | Chr09b | 20162137 | SNV | C | | G | | No | | 1 | Homozygous | 6487 | 6496 | 99.86 | 33.64 |
| Pavir.Ib01857.1 | Chr09b | 20162260 | SNV | C | | T | | No | | 1 | Homozygous | 3 | 3 | 100.00 | 40.33 |
| Pavir.Ib01857.1 | Chr09b | 20162266 | SNV | C | | G | | No | | 1 | Homozygous | 3 | 3 | 100.00 | 40.00 |
| Pavir.Ib01857.1 | Chr09b | 20162379..20162380 | Deletion | GG | | - | | No | | 2 | Homozygous | 12 | 12 | 100.00 | 38.92 |
| Pavir.Ib01857.1 | Chr09b | 20162574 | SNV | T | | G | | No | | 1 | Homozygous | 19 | 19 | 100.00 | 40.11 |
| Pavir.Ib01857.1 | Chr09b | 20162601 | SNV | T | | G | | No | | 1 | Homozygous | 29 | 29 | 100.00 | 38.83 |
| Pavir.Ib01857.1 | Chr09b | 20162626 | SNV | C | | T | | No | | 1 | Homozygous | 28 | 28 | 100.00 | 38.14 |
| Pavir.Ib01857.1 | Chr09b | 20162768 | SNV | A | | T | | No | | 1 | Heterozygous | 28 | 68 | 41.18 | 38.64 |
| Pavir.Ib01857.1 | Chr09b | 20162867 | SNV | C | | T | | No | | 1 | Homozygous | 17 | 17 | 100.00 | 39.47 |
| Pavir.Ib01857.1 | Chr09b | 20163135^20163136 | Insertion | T | | - | | No | | 1 | Homozygous | 4 | 5 | 80.00 | 41.00 |
| Pavir.Ib01857.1 | Chr09b | 20163139 | SNV | T | | C | | No | | 1 | Homozygous | 4 | 5 | 80.00 | 41.00 |
| Pavir.Ib01857.1 | Chr09b | 20163313 | SNV | C | | T | | No | | 1 | Homozygous | 55 | 55 | 100.00 | 40.20 |
| Pavir.Ib01857.1 | Chr09b | 20163848 | SNV | T | | C | | No | | 1 | Heterozygous | 1301 | 2834 | 45.91 | 39.52 |
| Pavir.Ib01857.1 | Chr09b | 20164127 | SNV | G | | A | | No | | 1 | Homozygous | 6 | 6 | 100.00 | 40.50 |
| Pavir.J1005.1 | contig01099 | 13673 | SNV | T | | C | | No | | 1 | Homozygous | 11 | 11 | 100.00 | 37.18 |
| Pavir.J1005.1 | contig01099 | 13683 | SNV | G | | A | | No | | 1 | Homozygous | 11 | 11 | 100.00 | 38.45 |
| Pavir.J1005.1 | contig01099 | 14144 | SNV | A | | T | | No | | 1 | Homozygous | 15 | 15 | 100.00 | 38.40 |
| Pavir.J1005.1 | contig01099 | 14154 | SNV | T | | G | | No | | 1 | Homozygous | 10 | 10 | 100.00 | 34.80 |
| Pavir.J1005.1 | contig01099 | 14267 | SNV | C | | T | | No | | 1 | Homozygous | 11 | 11 | 100.00 | 32.27 |
| Pavir.J1005.1 | contig01099 | 15817 | SNV | A | | G | | No | | 1 | Heterozygous | 4 | 13 | 30.77 | 41.00 |
| Pavir.J1005.1 | contig01099 | 15850 | SNV | G | | T | | No | | 1 | Homozygous | 4 | 4 | 100.00 | 40.25 |
| Pavir.J1005.1 | contig01099 | 15856 | SNV | G | | A | | No | | 1 | Homozygous | 3 | 3 | 100.00 | 41.00 |
| Pavir.J1005.1 | contig01099 | 15889..15890 | MNV | CA | | TG | | No | | 2 | Homozygous | 2 | 3 | 66.67 | 40.25 |
|  |  |  |  |  | |  | |  | |  |  |  |  |  |  |
| Pavir.J1005.1 | contig01099 | 15895 | SNV | C | | T | | No | | 1 | Homozygous | 3 | 4 | 75.00 | 40.33 |
| Pavir.J1005.1 | contig01099 | 17136 | SNV | A | | A | | Yes | | 1 | Heterozygous | 6 | 8 | 75.00 | 39.67 |
| Pavir.J1005.1 | contig01099 | 17136 | SNV | G | | A | | No | | 1 | Heterozygous | 2 | 8 | 25.00 | 40.50 |
| Pavir.J1005.1 | contig01099 | 17961 | SNV | G | | C | | No | | 1 | Heterozygous | 2 | 5 | 40.00 | 40.00 |
| Pavir.J1005.1 | contig01099 | 17966 | SNV | C | | T | | No | | 1 | Homozygous | 4 | 4 | 100.00 | 36.50 |
| Pavir.J05563.1 | contig08697 | 7556 | SNV | T | | C | | No | | 1 | Homozygous | 13 | 13 | 100.00 | 39.85 |
| Pavir.J05563.1 | contig08697 | 7557^7558 | Insertion | TA | | - | | No | | 2 | Heterozygous | 6 | 11 | 54.55 | 34.92 |
| Pavir.J05563.1 | contig08697 | 7578 | SNV | A | | T | | No | | 1 | Homozygous | 40 | 40 | 100.00 | 39.65 |
| Pavir.J05563.1 | contig08697 | 7607 | SNV | A | | G | | No | | 1 | Homozygous | 108 | 108 | 100.00 | 39.02 |
| Pavir.J05563.1 | contig08697 | 7629 | SNV | G | | T | | No | | 1 | Homozygous | 11 | 11 | 100.00 | 35.09 |
| Pavir.J05563.1 | contig08697 | 7657 | SNV | T | | C | | No | | 1 | Heterozygous | 2 | 4 | 50.00 | 38.00 |
| Pavir.J05563.1 | contig08697 | 7672 | SNV | A | | T | | No | | 1 | Homozygous | 45 | 45 | 100.00 | 39.07 |
| Pavir.J05563.1 | contig08697 | 7692 | SNV | A | | T | | No | | 1 | Heterozygous | 18 | 48 | 37.50 | 38.06 |
| Pavir.J05563.1 | contig08697 | 7699 | SNV | C | | A | | No | | 1 | Heterozygous | 30 | 68 | 44.12 | 38.37 |
| Pavir.J05563.1 | contig08697 | 7719^7720 | Insertion | - | | GGC | | No | | 3 | Homozygous | 57 | 57 | 100.00 | 34.49 |
| Pavir.J05563.1 | contig08697 | 7746^7747 | Insertion | T | | - | | No | | 1 | Heterozygous | 13 | 44 | 29.55 | 38.31 |
| Pavir.J05563.1 | contig08697 | 7759 | SNV | C | | A | | No | | 1 | Heterozygous | 72 | 122 | 59.02 | 40.11 |
| Pavir.J05563.1 | contig08697 | 7765 | SNV | C | | A | | No | | 1 | Heterozygous | 72 | 111 | 64.86 | 38.97 |
| Pavir.J05563.1 | contig08697 | 7772 | SNV | A | | G | | No | | 1 | Heterozygous | 19 | 26 | 73.08 | 35.42 |
| Pavir.J05563.1 | contig08697 | 7779 | SNV | G | | A | | No | | 1 | Heterozygous | 19 | 53 | 35.85 | 39.58 |
| Pavir.J05563.1 | contig08697 | 7818 | SNV | C | | G | | No | | 1 | Heterozygous | 9 | 34 | 26.47 | 40.89 |
| Pavir.J05563.1 | contig08697 | 7947 | SNV | G | | T | | No | | 1 | Heterozygous | 15 | 23 | 65.22 | 38.73 |
| Pavir.J05563.1 | contig08697 | 8140 | SNV | A | | C | | No | | 1 | Homozygous | 4 | 5 | 80.00 | 37.75 |
| Pavir.J05563.1 | contig08697 | 8166 | SNV | C | | A | | No | | 1 | Homozygous | 12 | 12 | 100.00 | 39.83 |
| Pavir.J05563.1 | contig08697 | 8203 | SNV | C | | A | | No | | 1 | Homozygous | 9 | 9 | 100.00 | 39.22 |
| Pavir.J05563.1 | contig08697 | 8207 | SNV | T | | A | | No | | 1 | Homozygous | 9 | 9 | 100.00 | 39.67 |
| Pavir.J05563.1 | contig08697 | 8326 | SNV | T | | C | | No | | 1 | Homozygous | 193 | 193 | 100.00 | 39.13 |
| Pavir.J05563.1 | contig08697 | 8368 | SNV | G | | A | | No | | 1 | Homozygous | 582 | 583 | 99.83 | 39.91 |
| Pavir.J05563.1 | contig08697 | 8389 | SNV | T | | C | | No | | 1 | Heterozygous | 16 | 44 | 36.36 | 38.75 |
| Pavir.J24812.1 | contig27272 | 4620 | SNV | T | | C | | No | | 1 | Homozygous | 7 | 7 | 100.00 | 40.29 |
| Pavir.J24812.1 | contig27272 | 4689 | SNV | A | | C | | No | | 1 | Heterozygous | 1164 | 2264 | 51.41 | 35.81 |
| Pavir.J24812.1 | contig27272 | 4716 | SNV | A | | T | | No | | 1 | Heterozygous | 326 | 728 | 44.78 | 36.46 |
| Pavir.J24812.1 | contig27272 | 4814 | Deletion | A | | - | | No | | 1 | Homozygous | 13 | 13 | 100.00 | 34.92 |
| Pavir.J24812.1 | contig27272 | 4900 | SNV | G | | A | | No | | 1 | Heterozygous | 6 | 20 | 30.00 | 37.17 |
| Pavir.J24812.1 | contig27272 | 4977 | SNV | C | | T | | No | | 1 | Homozygous | 15 | 15 | 100.00 | 39.53 |
| Pavir.J24812.1 | contig27272 | 5037 | SNV | G | | A | | No | | 1 | Homozygous | 20 | 20 | 100.00 | 40.50 |
| Pavir.J24812.1 | contig27272 | 5074..5075 | Deletion | TA | | - | | No | | 2 | Heterozygous | 2 | 8 | 25.00 | 39.00 |
| Pavir.J24812.1 | contig27272 | 5097 | SNV | T | | G | | No | | 1 | Homozygous | 6 | 6 | 100.00 | 40.00 |
| Pavir.J24812.1 | contig27272 | 5119..5120 | MNV | AC | | GT | | No | | 2 | Homozygous | 7 | 9 | 77.78 | 37.07 |
| Pavir.J24812.1 | contig27272 | 5262 | SNV | T | | G | | No | | 1 | Heterozygous | 7 | 15 | 46.67 | 40.14 |
| Pavir.J24812.1 | contig27272 | 5477 | SNV | T | | G | | No | | 1 | Heterozygous | 4 | 14 | 28.57 | 39.75 |
| Pavir.J24812.1 | contig27272 | 5477 | SNV | T | | T | | Yes | | 1 | Heterozygous | 10 | 14 | 71.43 | 38.30 |
| Pavir.J24812.1 | contig27272 | 5564 | SNV | T | | C | | No | | 1 | Heterozygous | 3 | 12 | 25.00 | 40.33 |
| Pavir.J24812.1 | contig27272 | 5564 | SNV | T | | T | | Yes | | 1 | Heterozygous | 9 | 12 | 75.00 | 39.67 |
| Pavir.J24812.1 | contig27272 | 5659 | SNV | T | | C | | No | | 1 | Heterozygous | 6 | 20 | 30.00 | 37.33 |
| Pavir.J24812.1 | contig27272 | 5695 | SNV | C | | T | | No | | 1 | Homozygous | 8 | 8 | 100.00 | 39.88 |
| Pavir.J24812.1 | contig27272 | 5795 | SNV | G | | A | | No | | 1 | Homozygous | 120 | 120 | 100.00 | 39.23 |
| Pavir.J24812.1 | contig27272 | 6158 | SNV | C | | C | | Yes | | 1 | Heterozygous | 3 | 6 | 50.00 | 39.67 |
| Pavir.J24812.1 | contig27272 | 6158 | SNV | T | | C | | No | | 1 | Heterozygous | 3 | 6 | 50.00 | 38.67 |
| Pavir.J24812.1 | contig27272 | 6205 | SNV | C | | T | | No | | 1 | Homozygous | 2 | 3 | 66.67 | 39.50 |
| Pavir.J24812.1 | contig27272 | 6224 | SNV | C | | T | | No | | 1 | Heterozygous | 2 | 4 | 50.00 | 40.50 |
| Pavir.J24812.1 | contig27272 | 6651 | SNV | G | | T | | No | | 1 | Heterozygous | 47 | 127 | 37.01 | 39.74 |
| Pavir.J24812.1 | contig27272 | 6766 | Replacement | C | | AA | | No | | 2 | Heterozygous | 11 | 29 | 37.93 | 40.23 |
| Pavir.J24812.1 | contig27272 | 6880 | SNV | A | | G | | No | | 1 | Heterozygous | 4 | 7 | 57.14 | 34.75 |
| Pavir.J26857.1 | contig30080 | 574 | SNV | T | | C | | No | | 1 | Homozygous | 5 | 5 | 100.00 | 39.80 |
| Pavir.J26857.1 | contig30080 | 668 | SNV | G | | T | | No | | 1 | Homozygous | 7 | 7 | 100.00 | 38.71 |
| Pavir.J26857.1 | contig30080 | 719 | SNV | C | | C | | Yes | | 1 | Heterozygous | 2 | 4 | 50.00 | 41.00 |
| Pavir.J26857.1 | contig30080 | 719 | SNV | T | | C | | No | | 1 | Heterozygous | 2 | 4 | 50.00 | 41.00 |
| Pavir.J26857.1 | contig30080 | 834 | SNV | A | | A | | Yes | | 1 | Heterozygous | 2 | 5 | 40.00 | 35.50 |
| Pavir.J26857.1 | contig30080 | 834 | SNV | C | | A | | No | | 1 | Heterozygous | 3 | 5 | 60.00 | 40.00 |
| Pavir.J26857.1 | contig30080 | 1851 | SNV | T | | A | | No | | 1 | Heterozygous | 36 | 106 | 33.96 | 39.19 |
| Pavir.J26857.1 | contig30080 | 1880 | SNV | T | | A | | No | | 1 | Heterozygous | 39 | 145 | 26.90 | 39.28 |
| Pavir.J26857.1 | contig30080 | 1893 | SNV | T | | C | | No | | 1 | Heterozygous | 23 | 70 | 32.86 | 37.48 |
| Pavir.J26857.1 | contig30080 | 2020 | SNV | T | | G | | No | | 1 | Heterozygous | 3 | 8 | 37.50 | 39.00 |
| Pavir.J26857.1 | contig30080 | 2032^2033 | Insertion | G | | - | | No | | 1 | Heterozygous | 9 | 19 | 47.37 | 38.89 |
| Pavir.J09299.1 | contig123174 | 171 | SNV | G | | A | | No | | 1 | Heterozygous | 11 | 42 | 26.19 | 38.55 |
| Pavir.J09299.1 | contig123174 | 175 | SNV | A | | G | | No | | 1 | Homozygous | 4 | 4 | 100.00 | 38.25 |
| Pavir.J09299.1 | contig123174 | 413 | SNV | C | | G | | No | | 1 | Homozygous | 4 | 4 | 100.00 | 41.00 |
| Pavir.J09299.1 | contig123174 | 476 | SNV | G | | A | | No | | 1 | Homozygous | 12 | 12 | 100.00 | 39.25 |
| Pavir.J09299.1 | contig123174 | 793 | SNV | T | | C | | No | | 1 | Homozygous | 6 | 6 | 100.00 | 40.17 |
| Pavir.J09299.1 | contig123174 | 795 | SNV | T | | C | | No | | 1 | Homozygous | 6 | 6 | 100.00 | 39.67 |
| Pavir.J09299.1 | contig123174 | 872 | SNV | C | | T | | No | | 1 | Homozygous | 4 | 4 | 100.00 | 40.00 |
| Pavir.J09299.1 | contig123174 | 895 | SNV | G | | A | | No | | 1 | Homozygous | 15 | 15 | 100.00 | 38.93 |
| Pavir.J09299.1 | contig123174 | 895 | SNV | T | | G | | No | | 1 | Homozygous | 10 | 10 | 100.00 | 38.90 |
| Pavir.J09299.1 | contig123174 | 1001 | SNV | G | | A | | No | | 1 | Homozygous | 11 | 11 | 100.00 | 39.73 |
| Pavir.J09299.1 | contig123174 | 1019^1020 | Insertion | - | | TG | | No | | 2 | Homozygous | 7 | 7 | 100.00 | 40.07 |
| Pavir.J09299.1 | contig123174 | 1044 | SNV | T | | C | | No | | 1 | Homozygous | 6 | 7 | 85.71 | 36.00 |
| Pavir.J09299.1 | contig123174 | 1103 | SNV | C | | G | | No | | 1 | Homozygous | 5 | 5 | 100.00 | 39.20 |
| Pavir.J09299.1 | contig123174 | 1108 | SNV | G | | A | | No | | 1 | Homozygous | 5 | 5 | 100.00 | 39.60 |
| Pavir.J09299.1 | contig123174 | 1188 | SNV | T | | C | | No | | 1 | Homozygous | 3 | 3 | 100.00 | 35.33 |
| Pavir.J09299.1 | contig123174 | 1359 | Deletion | - | | T | | No | | 1 | Homozygous | 3 | 3 | 100.00 | 37.33 |
| Pavir.J00640.1 | contig00653 | 13703 | SNV | C | | T | | No | | 1 | Heterozygous | 9 | 22 | 40.91 | 39.44 |
| Pavir.J00640.1 | contig00653 | 13703 | SNV | T | | T | | Yes | | 1 | Heterozygous | 13 | 22 | 59.09 | 38.08 |
| Pavir.J00640.1 | contig00653 | 13714 | SNV | A | | A | | Yes | | 1 | Heterozygous | 18 | 24 | 75.00 | 35.00 |
| Pavir.J00640.1 | contig00653 | 13714 | SNV | G | | A | | No | | 1 | Heterozygous | 6 | 24 | 25.00 | 38.67 |
| Pavir.J05674.2 | contig08989 | 10655 | SNV | T | | A | | No | | 1 | Homozygous | 4 | 4 | 100.00 | 39.50 |
| Pavir.J05674.2 | contig08989 | 10709 | SNV | T | | G | | No | | 1 | Homozygous | 3 | 3 | 100.00 | 39.67 |
| Pavir.J05674.2 | contig08989 | 10750^10751 | Insertion | - | | AC | | No | | 2 | Heterozygous | 4 | 6 | 66.67 | 38.75 |
| Pavir.J05674.2 | contig08989 | 12009 | SNV | T | | C | | No | | 1 | Heterozygous | 2 | 5 | 40.00 | 41.00 |
| Pavir.J05674.2 | contig08989 | 12661 | SNV | C | | G | | No | | 1 | Heterozygous | 5 | 8 | 62.50 | 35.20 |
| Pavir.J05674.2 | contig08989 | 13001..13002 | MNV | CC | | AT | | No | | 2 | Homozygous | 4 | 4 | 100.00 | 37.00 |
| Pavir.J05674.2 | contig08989 | 13005 | SNV | A | | G | | No | | 1 | Homozygous | 4 | 4 | 100.00 | 41.00 |
| Pavir.J05674.2 | contig08989 | 13041 | SNV | G | | T | | No | | 1 | Homozygous | 3 | 4 | 75.00 | 39.00 |
